# Opioidergic pathways and kisspeptin in the regulation of female reproduction in mammals

**DOI:** 10.3389/fnins.2022.958377

**Published:** 2022-08-11

**Authors:** Yoshihisa Uenoyama, Hitomi Tsuchida, Mayuko Nagae, Naoko Inoue, Hiroko Tsukamura

**Affiliations:** Laboratory of Animal Reproduction, Graduate School of Bioagricultural Sciences, Nagoya University, Nagoya, Japan

**Keywords:** endogenous opioid peptides, dynorphin, β-endorphin, enkephalin, GnRH pulse generator, GnRH surge generator, KNDy neurons, *Kiss1*

## Abstract

Endogenous opioid peptides have attracted attention as critical neuropeptides in the central mechanism regulating female reproduction ever since the discovery that arcuate dynorphin neurons that coexpress kisspeptin and neurokinin B (NKB), which are also known as kisspeptin/neurokinin B/dynorphin (KNDy) neurons, play a role as a master regulator of pulsatile gonadotropin-releasing hormone (GnRH) release in mammals. In this study, we first focus on the role of dynorphin released by KNDy neurons in the GnRH pulse generation. Second, we provide a historical overview of studies on endogenous opioid peptides. Third, we discuss how endogenous opioid peptides modulate tonic GnRH/gonadotropin release in female mammals as a mediator of inhibitory internal and external cues, such as ovarian steroids, nutritional status, or stress, on reproduction. Then, we discuss the role of endogenous opioid peptides in GnRH surge generation in female mammals.

## Introduction

One of the most important findings on the role of endogenous opioid peptides in female reproduction over the last two decades is that dynorphin neurons in the hypothalamic arcuate nucleus (ARC) are involved in gonadotropin-releasing hormone (GnRH) pulse generation. More specifically, a majority of ARC dynorphin neurons coexpress kisspeptin and neurokinin B (NKB); thus, the neurons are also referred to as kisspeptin/neurokinin B/dynorphin (KNDy) neurons and act as master regulators of pulsatile GnRH release in mammals (Lehman et al., 2010a; Maeda et al., 2010; Okamura et al., 2013; Uenoyama et al., 2014, 2021b; Goodman et al., 2018; Moore et al., 2018; Ikegami et al., 2021; Nagae et al., 2021; Tsukamura, 2022). GnRH is intermittently secreted in the pituitary portal vessel (Clarke and Cummins, 1982; Moenter et al., 1992) and controls the tonic (pulsatile) release of luteinizing hormone (LH) and follicle-stimulating hormone (FSH) from the anterior lobe of the pituitary gland. The tonic release of LH and FSH governs follicular development and corpus luteum function in the ovaries of female mammals. GnRH pulses are fundamental for reproduction in female mammals as a pioneer study demonstrated that circulating LH and FSH levels were maintained only when GnRH was applied in a pulsatile manner at physiological intervals in female rhesus monkeys after the blockade of endogenous GnRH release by a hypothalamic lesion (Belchetz et al., 1978). The neuronal circuit driving GnRH pulse generation has generally been termed the GnRH pulse generator (Lincoln et al., 1985; Maeda et al., 1995), and the intrinsic source of the generator has been a major enigma until very recently.

The present review mainly focuses on how endogenous opioid peptides regulate and/or modulate tonic GnRH/gonadotropin release, which is regulated by KNDy neurons, in female mammals. We also provide a historical overview of studies on endogenous opioid peptides and a summary of our recent understanding of the role of hypothalamic opioidergic neurons in the mechanism regulating female reproduction under normal and stressful conditions.

## Kisspeptin/neurokinin B/dynorphin neurons as an intrinsic regulator of gonadotropin-releasing hormone pulses

Since the discovery of KNDy neurons, endogenous opioid peptides have attracted attention as critical neuropeptides in the central mechanism regulating female reproduction. Indeed, the discovery of KNDy neurons is one of the most exciting topics in reproductive neuroendocrinology over the last two decades. Using immunohistochemistry, Goodman and colleagues first demonstrated that dynorphin and NKB are largely coexpressed in a single population of ARC neurons in ewes (Foradori et al., 2006) and then revealed that kisspeptin is also expressed in the majority of the same neuronal population (Goodman et al., 2007). Importantly, Goodman and colleagues reported that none of the dynorphin neuronal populations located in the other hypothalamic regions, such as the paraventricular nucleus (PVN), supraoptic nucleus (SON), and preoptic area (POA), colocalized with NKB (Foradori et al., 2006) and kisspeptin (Goodman et al., 2007). Immediately thereafter, the coexpression of dynorphin, NKB, and kisspeptin in a population of ARC neurons was validated in several mammals, including goats (Wakabayashi et al., 2010), heifers (Hassaneen et al., 2016), rats (True et al., 2011; Murakawa et al., 2016), mice (Navarro et al., 2009; Ikegami et al., 2017), pigs (Harlow et al., 2021), and rhesus monkeys (Ramaswamy et al., 2010; True et al., 2017), as summarized in our recent article (Uenoyama et al., 2021b). These findings implied the physiological importance of KNDy neurons for mammalian reproduction beyond the species, although colocalization of dynorphin in ARC kisspeptin/NKB neurons was not evident yet in humans (Hrabovszky et al., 2012, 2019).

**FIGURE 1:**
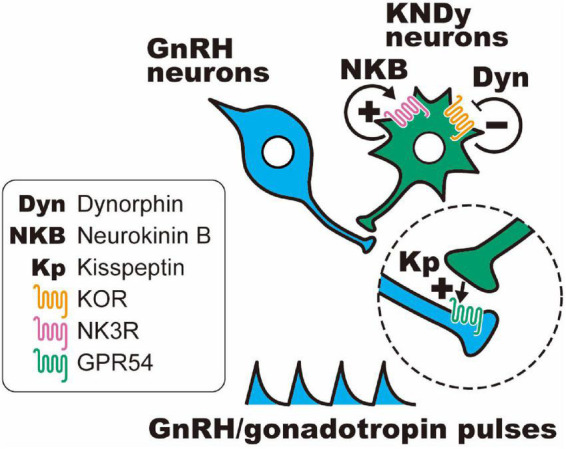
Schematic illustration of the hypothetical mechanism of gonadotropin-releasing hormone (GnRH) pulse generation in female mammals. Dynorphin (Dyn) released from KNDy neurons arrests KNDy neuronal activity *via* inhibitory Gi/o-coupled κ-opioid receptors (KORs), and neurokinin B (NKB) initiates synchronized KNDy neuronal activity *via* stimulatory Gq-coupled NKB receptors (also known as NK3R) to release kisspeptin, and kisspeptin, in turn, stimulates GnRH release *via* stimulatory Gq-coupled kisspeptin receptors (also known as GPR54) expressed in GnRH neurons.

Importantly, dynorphin receptors (i.e., κ-opioid receptors; KORs) were found in a majority of rat and ovine KNDy neurons (Weems et al., 2016; Tsuchida et al., 2020) and a portion of KNDy neurons in female mice (Navarro et al., 2009; Ikegami et al., 2017). In addition, the NKB receptors (also known as NK3R) were found in a majority of rodent and ovine KNDy neurons (Navarro et al., 2009; Amstalden et al., 2010; Ikegami et al., 2017). On the other hand, kisspeptin receptors (also known as GPR54) were found in the majority of GnRH neurons and were scarcely found in KNDy neurons of mice and rats (Herbison et al., 2010; Higo et al., 2016). These findings suggest that KNDy neurons communicate with each other by dynorphin-KOR and NKB-NK3R signaling in an autocrine/paracrine manner. As shown in Figure 1, the most plausible interpretation of the cellular mechanism regulating synchronized KNDy neuronal activity to drive GnRH pulses is as follows: we envisage that dynorphin released from KNDy neurons arrests KNDy neuronal activity *via* the inhibitory Gi/o-coupled KOR, NKB initiates synchronized KNDy neuronal activity *via* stimulatory Gq-coupled NK3R to release kisspeptin, and kisspeptin, in turn, stimulates GnRH release *via* stimulatory Gq-coupled GPR54 expressed in GnRH neurons (Navarro et al., 2009; Lehman et al., 2010a,b; Okamura et al., 2013; Uenoyama et al., 2014, 2021b; Goodman et al., 2018; Moore et al., 2018; Ikegami et al., 2021). Indeed, in female goats, the frequency of multiple unit activity (MUA) volleys, which were recorded in the vicinity of ARC KNDy neurons and accompanied by LH pulses, was decreased by the central administration of dynorphin and increased by the administration of a KOR antagonist (nor-binaltorphimine; nor-BNI) or NKB (Ohkura et al., 2009; Wakabayashi et al., 2010). These findings suggest that dynorphin-KOR signaling and NKB-NK3R signaling play a role in determining the frequency of GnRH pulse generator activities. Furthermore, central or peripheral administration of dynorphin or NK3R antagonists (SB223412 and SB222200) suppressed LH pulses, whereas KOR antagonists (nor-BNI and PF-4455242); NKB, an NK3R agonist (senktide); and kisspeptin stimulated LH pulses in several mammalian species, such as rodents (Gottsch et al., 2004; Irwig et al., 2004; Kinoshita et al., 2005; Messager et al., 2005; Pheng et al., 2009; Navarro et al., 2011; Mostari et al., 2013; Ruiz-Pino et al., 2015) and ruminants (Messager et al., 2005; Ohkura et al., 2009; Sakamoto et al., 2012; Tanaka et al., 2012; Goodman et al., 2013; Naniwa et al., 2013; Yamamura et al., 2015; Nakamura et al., 2017; Sasaki et al., 2019, 2020).

Recently, we rescued *Kiss1* (which is the gene that encodes kisspeptin) expression in ARC dynorphin/NKB neurons in global *Kiss1*-knockout rats utilizing adeno-associated virus (AAV) vectors carrying *Kiss1* cDNA (Nagae et al., 2021). Rescuing *Kiss1* expression in 20–50% of ARC NKB neurons could recover pulsatile LH release and folliculogenesis up to the preovulatory follicles in global *Kiss1*-knockout female rats. These findings provide direct evidence that ARC KNDy neurons serve as an intrinsic source of the GnRH pulse generator in female mammals.

## Brief history of studies on endogenous opioid peptides

**FIGURE 2:**
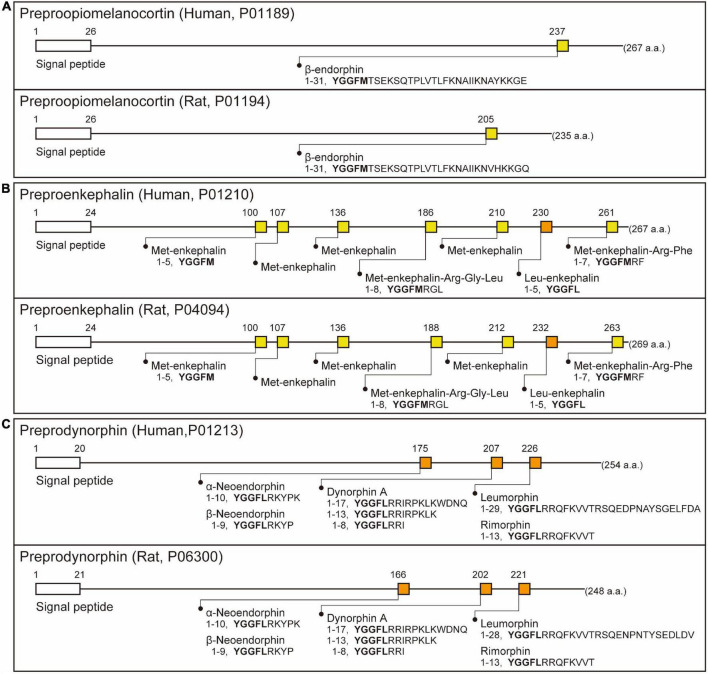
Schematic illustration of β-endorphin, Met- and Leu-enkephalins, and the dynorphin family (dynorphin A, α- and β-neoendorphins, leumorphin, and rimorphin) in their precursors in humans and rats based on UniProtKB (https://www.uniprot.org/uniprot/). The precursors comprise a signal peptide at the N-terminal. **(A)** β-Endorphin consists of 31 amino acids cleaved from the precursor preproopiomelanocortin in humans and rats. Note that the five N-terminal amino acids (YGGMF, yellow squares) of β-endorphin, identical to Met-enkephalin, are commonly found in the mammals examined. **(B)** Met- (YGGMF, yellow squares) and Leu-enkephalins (YGGML, orange squares) consist of five amino acids cleaved from the precursor preproenkephalin. Note that human and rat preproenkephalin possess six Met-enkephalin and one Leu-enkephalin motifs, and two of six Met-enkephalin motifs are processed to eight or seven amino acid peptides (Met-enkephalin-Arg-Gly-Leu and Met-enkephalin-Arg-Phe). **(C)** Dynorphin A, α- and β-neoendorphins, leumorphin, and rimorphin (also known as dynorphin B) consist of 8–28 amino acids cleaved from the single precursor preprodynorphin. Note that the five N-terminal amino acids (YGGML, orange squares) of all dynorphin family peptides are identical to Leu-enkephalin. The amino acid sequence of dynorphin A is identical among the mammals examined.

Endogenous opioid peptides were found to be endogenous substances that produce the same analgesic effect as morphine, an opiate alkaloid derived from opium poppies (Brownstein, 1993; Snyder and Pasternak, 2003; Waldhoer et al., 2004; Gruber et al., 2007; Przewlocki, 2013; Devereaux et al., 2018). Opiate alkaloids have a long history of medicinal use since the time of ancient Greeks and Romans (Brownstein, 1993; Waldhoer et al., 2004; Gruber et al., 2007; Devereaux et al., 2018), and the active ingredient morphine was isolated in the middle of the 1800s (Devereaux et al., 2018). Morphine was introduced for pain treatment in the 1820s (Przewlocki, 2013; Devereaux et al., 2018), and then morphine, like original opiate alkaloids, was found to be an addictive drug (Brownstein, 1993; Przewlocki, 2013). In search of a safe analgesic, many opiate agonists and antagonists were developed (Brownstein, 1993; Gruber et al., 2007), and by the middle of the 1960s, it was becoming clear that the analgesic effect of morphine and opiate agonists could be explained by the presence of specific receptors for the opiates in the brain (Snyder and Pasternak, 2003; Devereaux et al., 2018). In 1973, a radioreceptor assay with tritium-labeled and non-labeled opiate agonists or antagonists (Table 1) revealed the stereospecific binding of opiates, namely, opiate or morphine receptors, in rat brain homogenates (Pert and Snyder, 1973; Simon et al., 1973; Terenius, 1973). These findings implied the presence of endogenous opioidergic ligand(s) as neurotransmitters in the central nervous systems of mammals. In 1975, two pentapeptides, Tyr-Gly-Gly-Phe-Met (termed Met-enkephalin) and Tyr-Gly-Gly-Phe-Leu (termed Leu-enkephalin), were found in the pig brain as endogenous ligands for opiate or morphine receptors (Hughes et al., 1975a,b). It soon became obvious that the Met-enkephalin sequence was present on the N terminus of another endogenous opioid peptide, that is, β-endorphin, in 1976 (Birdsall and Hulme, 1976; Li and Chung, 1976). Subsequently, the Leu-enkephalin sequence was found at the N terminus of another endogenous opioid peptide, dynorphin, in 1979 (Goldstein et al., 1979, 1981). To date, these endogenous opioid peptides have been classified into three families and were reported to be derived from three distinct precursors encoded by *Pomc*, *Penk*, and *Pdyn* genes (Nakanishi et al., 1979; Kakidani et al., 1982; Noda et al., 1982; Akil et al., 1984; Froehlich, 1997; Benarroch, 2012). Figure 2 shows three precursors—preproopiomelanocortin, preproenkephalin, and preprodynorphin—of endogenous opioid peptides, such as β-endorphin, Met- and Leu-enkephalins, and the dynorphin family [dynorphin A, and α- and β-neoendorphins, leumorphin, and rimorphin (also known as dynorphin B)], respectively, in humans and rats.

**TABLE 1:** Representative opiate agonists and antagonists used in the radioreceptor assay.

References	Agonists	Antagonists
Pert and Snyder, 1973	Codeine	Naloxone
	Levorphanol	Levallorphan
	Methadone	Nalorphine
	Morphine	
	Pentazocine*	
	Propoxyphene	
Simon et al., 1973	Etorphine	Nalorphine
	Levorphanol	Naloxone
	Methadone	
	Morphine	
Terenius, 1973	Codeine	Naloxone
	Dihydromorphine	
	Heroin	
	Levorphanol	

*Partial agonist.

It is well known that β-endorphin, Met- and Leu-enkephalins, and the dynorphin family share three types of opioid receptors—μ-, δ-, and κ-opioid receptors (MOR, DOR, and KOR)—encoded by *Oprm1*, *Oprd1*, and *Oprk1* genes, respectively (Snyder and Pasternak, 2003; Waldhoer et al., 2004; Stein, 2016). As shown in Table 2, β-endorphin has been reported to predominantly bind to both MOR and DOR with a similar affinity and with a lower affinity for KOR; Met- and Leu-enkephalins predominantly bind to the DOR with much higher affinity than the MOR and KOR; all dynorphin family peptides predominantly bind to the KOR, rather than the MOR and DOR (Yasuda et al., 1993; Raynor et al., 1994; Mansour et al., 1995b). We should note that morphine was reported to predominantly bind to the MOR, followed by the KOR, with a low affinity for the DOR (Mansour et al., 1995b). These opioid receptors were cloned in rats and mice during the early 1990s (Evans et al., 1992; Kieffer et al., 1992; Chen et al., 1993a,b; Fukuda et al., 1993; Li et al., 1993; Minami et al., 1993; Nishi et al., 1993; Thompson et al., 1993) and were found to belong to the large superfamily of seven-transmembrane G protein-coupled receptors. After the binding of an agonist, conformational changes of all three opioid receptors predominantly allow intracellular coupling of a heterotrimeric Gi/o protein (Connor and Christie, 1999; Waldhoer et al., 2004; Stein, 2016). Therefore, opioid receptor activation leads to inhibited adenylyl cyclase activity and reduced cAMP levels in target neurons (Connor and Christie, 1999; Waldhoer et al., 2004; Stein, 2016). In addition, opioid receptor activation leads to the opening of G protein-coupled inwardly rectifying K^+^ channels, thereby preventing neuronal excitation and/or propagation of action potentials of target neurons (Connor and Christie, 1999; Waldhoer et al., 2004; Stein, 2016). From these findings, it is well accepted that endogenous opioid peptides serve as inhibitory signals in the central nervous system *via* inhibitory Gi/o-coupled opioid receptors in mammals.

**TABLE 2:** Binding affinity and specificity of morphine and endogenous opioid peptides to opioid receptors.

Agents	Specificity	Species	References
Morphine	μ- >>> κ- ≥ δ-	Mouse/rat^1^	Raynor et al., 1994
	μ- >> κ- >> δ-	Rat^2^	Mansour et al., 1995b
β-endorphin	δ- > κ-	Mouse^3^	Yasuda et al., 1993
	μ- ≥ δ- >>> κ-	Mouse/rat^1^	Raynor et al., 1994
	μ- ≥ δ- >> κ-	Rat^2^	Mansour et al., 1995b
Met-enkephalin	δ- >>> κ-	Mouse^3^	Yasuda et al., 1993
	μ- > δ- >>> κ-	Mouse/rat^1^	Raynor et al., 1994
	δ- > μ- >>> κ-	Rat^2^	Mansour et al., 1995b
Leu-enkephalin	δ- >>> κ-	Mouse^3^	Yasuda et al., 1993
	μ- ≥ δ- >>> κ-	Mouse/rat^1^	Raynor et al., 1994
	δ- >>> μ- >>> κ-	Rat^2^	Mansour et al., 1995b
Dynorphin A_1–17_	κ- >> δ-	Mouse^3^	Yasuda et al., 1993
	κ- > μ- > δ-	Mouse/rat^1^	Raynor et al., 1994
	κ- > δ- > μ-	Rat^2^	Mansour et al., 1995b
α-neoendorphin	κ- >>> δ-	Mouse^3^	Yasuda et al., 1993
	κ- >> μ- ≥ δ-	Rat^2^	Mansour et al., 1995b
β-neoendorphin	κ- > δ- > μ-	Rat^2^	Mansour et al., 1995b
Leumorphin	κ- >> δ- ≥ μ-	Rat^2^	Mansour et al., 1995b
Rimorphin	κ- >>> δ-	Mouse^3^	Yasuda et al., 1993
	κ- > μ- ≥ δ-	Rat^2^	Mansour et al., 1995b

>>>, more than 10 times; >>, more than 5 times; >, more than 2 times; ≥, less than 2 times.

^1^Cloned mouse DOR and KOR and rat MOR cDNA were examined.

^2^Cloned rat MOR, DOR, and KOR cDNA were examined.

^3^Cloned mouse MOR, DOR, and KOR cDNA were examined.

## Inhibitory roles of endogenous opioid peptides on tonic gonadotropin-releasing hormone/gonadotropin-releasing systems

Immediately after the isolation and characterization of the endogenous opioid peptides, the inhibitory effect of endogenous opioid peptides on pulsatile GnRH/gonadotropin release was intensively studied using the opioid receptor antagonist naloxone as a probe. As mentioned later in detail, peripheral and central administration of naloxone or other opioid antagonists facilitated tonic (pulsatile) LH release in female mammals at several stages of the reproductive cycle (Table 3) and under stressful conditions such as malnutrition and infection (Table 4). Thus, we envision that opioidergic neurons serve as mediators of inhibitory internal and external cues, such as ovarian steroids, nutritional status, or stress, on tonic GnRH/gonadotropin release in female mammals.

**TABLE 3:** Effects of opioid receptor antagonists on tonic luteinizing hormone (LH) secretion in female mammals.

Antagonists	Receptors	Treatment routes	Effects	Species	Ovarian states	References
Naloxone	μ- > κ- >> δ-	i.v.	Stimulatory	Human	Late follicular phase	Quigley and Yen, 1980
		i.v.	Stimulatory	Human	Mid-luteal phase	Quigley and Yen, 1980
		i.v.	No effect	Human	Early follicular phase	Quigley and Yen, 1980
		i.v.	Stimulatory	Rhesus monkey	Luteal phase	Van Vugt et al., 1983
		i.v.	No effect	Rhesus monkey	Follicular phase	Van Vugt et al., 1983
		s.c.	Stimulatory	Rat	Ovary-intact	Petraglia et al., 1984
		s.c.	No effect	Rat	OVX	Petraglia et al., 1984
		i.v.	Stimulatory	Sheep	Luteal phase	Malven et al., 1984
		i.v.	No effect	Sheep	Non-luteal phase	Malven et al., 1984
		i.v.	Stimulatory	Sheep	Early and mid-luteal phase	Brooks et al., 1986
		i.v.	No effect	Sheep	Late-luteal phase	Brooks et al., 1986
		i.v.	No effect	Human	Post-menopausal	Reid et al., 1983
		3V	Stimulatory	Rat	Pregnant	Gallo, 1990
		MBH, POA	Stimulatory	Sheep	Luteal phase	Goodman et al., 2004
WIN44,441-3	κ-	i.v.	Stimulatory	Sheep	Luteal phase	Whisnant and Goodman, 1988
		i.v.	No effect	Sheep	Follicular phase	Whisnant and Goodman, 1988
		i.v.	Stimulatory	Sheep	Luteal phase	Yang et al., 1988
nor-BNI	κ-	3V	Stimulatory	Rat	Pregnant	Gallo, 1990
		3V	Stimulatory	Rat	OVX + low E2^1^	Mostari et al., 2013
		3V	No effect	Rat	OVX	Mostari et al., 2013
		MBH, POA	Stimulatory	Sheep	Luteal phase	Goodman et al., 2004
PF-4455242	κ-	i.v., s.c.	Stimulatory	Goat	OVX + low E2^2^	Sasaki et al., 2019
Naloxonazine	μ-	POA	Stimulatory	Sheep	Luteal phase	Goodman et al., 2004
		MBH	No effect	Sheep	Luteal phase	Goodman et al., 2004
ICI 174864	δ-	3V	No effect	Rat	Pregnant	Gallo, 1990
Naltrindole	δ-	MBH, POA	No effect	Sheep	Luteal phase	Goodman et al., 2004

nor-BNI, nor-binaltorphimine.

^1^Ovariectomized (OVX) rats treated with a diestrous level of E2.

^2^OVX goats treated with a luteal phase level of E2.

**TABLE 4:** Effects of opioid receptor antagonists on tonic luteinizing hormone (LH) secretion in female mammals under stressful conditions.

Antagonist	Receptors	Effects	Treatments	Species	Ovarian states	References
Naloxone	μ- > κ- >> δ-	Restored	Electric shock stress	Rat	Proestrus	Hulse and Coleman, 1983
		Restored	120-h fasting	Rat	OVX	Dyer et al., 1985
		Restored	48-h fasting	Rat	OVX + low E2^1^	Cagampang et al., 1991
		Restored	hypoglycemia by insulin	Sheep	OVX	Clarke et al., 1990
		Restored	lipopolysaccharide	Cattle	OVX	Kujjo et al., 1995
		Restored	lipopolysaccharide	Rhesus monkey	OVX	Xiao et al., 2000
		Restored	CRH	Rhesus monkey	OVX	Gindoff and Ferin, 1987
		Restored	AVP	Rhesus monkey	OVX	Xiao et al., 1996
		Restored	CGRP	Rat	OVX	Bowe et al., 2005
β-funaltrexamine	μ-	Restored	CRH	Rat	OVX	Rivest et al., 1993
Naloxonazine	μ-	Restored	CRH	Rat	OVX	Rivest et al., 1993
CTOP	μ-	Restored	Glucoprivation by 2DG	Rat	OVX + low E2^1^	Tsuchida et al., 2021
nor-BNI	κ-	No effect	CRH	Rat	OVX	Rivest et al., 1993
		Restored	Glucoprivation by 2DG	Rat	OVX + low E2^1^	Tsuchida et al., 2020

CTOP, D-Phe-Cys-Tyr-D-Trp-Orn-Thr-Pen-Thr-NH_2_; CRH, corticotropin-releasing hormone; AVP, Arg-vasopressin; CGPR, calcitonin gene-related peptide; 2DG, 2-deoxy-D-glucose.

^1^OVX rats treated with a diestrous level of E2.

### Involvement of endogenous opioid peptides in mediating the negative feedback action of ovarian steroids on tonic gonadotropin-releasing hormone/gonadotropin release

It is well established that the frequency of GnRH/gonadotropin pulses is fine-tuned by the negative feedback action of ovarian steroids such as estradiol-17β (E2) and progesterone (P4) to keep circulating LH and FSH at proper levels to promote follicular development in the follicular phase of the estrous/menstrual cycle and maintain corpus luteum function in the luteal phase and pregnancy period (Nishihara et al., 1999; Herbison, 2020; Uenoyama et al., 2021a). Endogenous opioid peptides are suggested to be mediators of the negative feedback action of gonadal steroids on tonic GnRH/gonadotropin release in female mammals (summarized in Table 3). An intravenous (IV) injection of naloxone increased plasma LH levels during the late follicular (E2 dominant) and mid-luteal (P4 dominant) phases of the menstrual cycle, but not during the early follicular phase, in humans (Quigley and Yen, 1980). In addition, an IV injection of naloxone increased serum LH levels during the luteal phase, but not the follicular phase, in rhesus monkeys (Van Vugt et al., 1983). Likewise, subcutaneous (SC) injection of naloxone increased plasma LH levels in ovary-intact rats (Petraglia et al., 1984). An IV injection of naloxone stimulated LH secretion during the luteal phase, but not during the non-luteal phase, in ewes (Malven et al., 1984; Brooks et al., 1986). Furthermore, it has been noted that IV or SC administration of naloxone was unable to increase plasma LH levels in post-menopausal women (Reid et al., 1983) and ovariectomized (OVX) rats (Petraglia et al., 1984). In addition, naloxone administration into the third cerebroventricle (3V) facilitated LH pulses in rats during pregnancy (Gallo, 1990). These findings suggest that endogenous opioid peptides mediate the negative feedback action of E2 and P4 on pulsatile GnRH/LH release in female mammals. Furthermore, the local implant of crystalline naloxone into the mediobasal hypothalamus (MBH) or POA facilitated pulsatile LH release during the luteal phase in ewes (Goodman et al., 2004), suggesting that the MBH and POA, in which KNDy and GnRH neurons were found, respectively, in ewes (Lehman et al., 1986, 2010b; Goodman et al., 2007), could be possible action sites of endogenous opioid peptides to exert the negative feedback action of ovarian steroids on tonic GnRH/gonadotropin release. The expression of opioid receptors in KNDy and GnRH neurons will be discussed later.

Both KOR and MOR signaling are considered to mediate the negative feedback action of ovarian steroids on GnRH/gonadotropin release in female mammals. An IV injection of WIN44,441-3 (a specific KOR antagonist) facilitated LH pulses during the luteal phase of the estrous cycle (Whisnant and Goodman, 1988; Yang et al., 1988) but failed to facilitate LH pulses during the follicular phase in ewes (Whisnant and Goodman, 1988). Likewise, a 3V injection of nor-BNI (another KOR antagonist), but not ICI 174864 (a specific DOR antagonist), facilitated LH pulses in pregnant rats (Gallo, 1990). Our previous study showed that a 3V injection of nor-BNI stimulated the baseline levels of LH pulses in OVX rats treated with a diestrous level of E2, but not in OVX rats (Mostari et al., 2013). In addition, IV and SC injections of PF-4455242 (another KOR antagonist) facilitated LH pulses in OVX goats treated with a luteal phase level of E2 (Sasaki et al., 2019). Furthermore, the local implant of crystalline nor-BNI into the MBH or POA and the local implant of crystalline naloxonazine (a specific MOR antagonist) in the POA facilitated pulsatile LH release during the luteal phase in ewes (Goodman et al., 2004). By contrast, the local implant of crystalline naltrindole (a specific DOR antagonist) failed to facilitate pulsatile LH release during the luteal phase in ewes (Goodman et al., 2004). These results are consistent with the finding that naloxone was reported to predominantly bind to the MOR, followed by the KOR, with a low affinity for the DOR (Mansour et al., 1995b). Taken together, these findings suggest that endogenous opioid peptides may mediate the negative feedback action of ovarian steroids *via* KOR signaling in the MBH and KOR and MOR signaling in the POA in female mammals.

### Involvement of endogenous opioid peptides in mediating stress-induced suppression of tonic gonadotropin-releasing hormone/gonadotropin release

The frequency of GnRH/LH pulses is often suppressed under stressful conditions, such as malnutrition and infection (Chatterton, 1990; Tilbrook et al., 2000, 2002). Endogenous opioid peptides have attracted attention as mediators of the stress-induced suppression of GnRH/gonadotropin release in female mammals (summarized in Table 4). Previous studies have demonstrated that peripheral administration of naloxone blocks stress-induced LH suppression in several female mammals (Hulse and Coleman, 1983; Dyer et al., 1985; Clarke et al., 1990; Cagampang et al., 1991; Kujjo et al., 1995; Xiao et al., 2000). Concretely, an IV injection of naloxone blocked electric shock stress-induced LH suppression in proestrous female rats (Hulse and Coleman, 1983). Subcutaneous injections of naloxone blocked 48-h fasting-induced LH suppression in ovary-intact (Dyer et al., 1985) and OVX rats treated with a diestrous level of E2 (Cagampang et al., 1991). Our recent studies showed that the 3V administration of D-Phe-Cys-Tyr-D-Trp-Orn-Thr-Pen-Thr-NH2 (CTOP, another MOR antagonist) or nor-BNI restored suppression of LH pulses induced by peripheral or central injection of 2-deoxy-D-glucose (2DG, an inhibitor of glucose utilization) in OVX rats treated with a diestrus level of E2 (Tsuchida et al., 2020, 2021). Furthermore, IV administration of naloxone restored LH pulses that were suppressed by insulin-induced hypoglycemia in OVX ewes (Clarke et al., 1990), and IV injections of naloxone restored LH pulses that were suppressed by the administration of an endotoxin lipopolysaccharide in OVX heifers (Kujjo et al., 1995) and OVX rhesus monkeys (Xiao et al., 2000). Taken together, these findings suggest that endogenous opioid peptides mediate acute stress-induced suppression of GnRH/LH pulses under stressful conditions, such as malnutrition and infection, in female mammals.

It is well known that the stress response is mainly driven by the hypothalamic–pituitary–adrenal axis in mammals (Brooks and Challis, 1989; Senn et al., 1995; Bale and Vale, 2004; Papadimitriou and Priftis, 2009). Accumulating evidence has demonstrated that both corticotropin-releasing hormone (CRH) and Arg-vasopressin (AVP) neurons located in the PVN govern pituitary corticotrophin release and adrenal functions in response to various stressors (Brooks and Challis, 1989; Senn et al., 1995; Bale and Vale, 2004; Papadimitriou and Priftis, 2009). Thus, administration of CRH and AVP has been used to mimic stressful conditions to determine the role of opioids as mediators. As shown in Table 4, IV and lateral ventricle (LV) administration of naloxone restored CRH- and AVP-induced suppression of the frequency of LH pulses in OVX rhesus monkeys, respectively (Gindoff and Ferin, 1987; Xiao et al., 1996). These findings suggest that opioidergic signaling may mediate CRH/AVP-induced suppression of tonic GnRH/gonadotropin release in female mammals. Specifically, administration of β-funaltrexamine and naloxonazine (specific MOR antagonists), but not nor-BNI (a KOR antagonist), into the POA partially restored CRH-induced LH suppression in OVX rats (Rivest et al., 1993), suggesting that MOR signaling mainly mediates the suppression. In addition, O’Byrne and colleagues (Bowe et al., 2005) demonstrated that the LV injection of naloxone restored LH pulses that were suppressed by LV administration of calcitonin gene-related peptide, another mediator of stress-induced LH suppression (Li et al., 2004), in OVX rats.

### Involvement of endogenous opioid peptides in mediating pre-pubertal restraints of tonic gonadotropin-releasing hormone/gonadotropin release

It has been established that pre-pubertal restraints of GnRH/gonadotropin pulses are tightly associated with the negative feedback action of estrogen in rats and sheep (Foster and Ryan, 1979; Uenoyama et al., 2019). Endogenous opioid peptides are likely to mediate the estrogen-dependent pre-pubertal restraint of tonic GnRH/gonadotropin release in female mammals (summarized in Table 5). Ieiri et al. (1980) showed that SC administration of naloxone increased serum LH levels in pre-pubertal female rats. Ebling et al. (1989) suggested that endogenous opioidergic signaling mediates the estrogen-negative feedback action on pre-pubertal restraints of GnRH/gonadotropin pulses in lambs because IV administration of naloxone stimulated LH pulses in ovary-intact and E2-treated pre-pubertal OVX lambs. Ebling et al. (1989) also reported that naloxone was able to further increase the frequency of LH pulses shown in OVX pre-pubertal lambs in this study. Similarly, Wood et al. (1992) showed that naloxone was able to stimulate LH pulses in OVX pre-pubertal lambs in an estrogen-dependent manner. Furthermore, Nakahara et al. (2013) showed that chronic intraperitoneal infusion of nor-BNI increased LH pulses and hence advanced puberty onset in ovary-intact female rats. Similarly, Lopez et al. (2016) showed that LV infusion of non-BNI stimulated LH pulses in pre-pubertal E2-treated OVX lambs. Taken together, these findings suggest that central opioidergic signaling, at least KOR signaling, mediates the estrogen-dependent restraint of GnRH/gonadotropin pulses during the pre-pubertal period and may serve as a key determinant of puberty onset, at least in rats and sheep. It should be noted that Lopez et al. (2016) also showed that E2 replacement failed to increase dynorphin immunoreactivity in the ARC of pre-pubertal lambs, although P4 replacement increased dynorphin immunoreactivity in the ARC of post-pubertal female sheep. Thus, non-ARC dynorphin neurons may play a key role in the pre-pubertal restraint of GnRH/gonadotropin pulses in female sheep.

**TABLE 5:** Effects of opioid receptor antagonists on tonic luteinizing hormone (LH) secretion in pre-pubertal female mammals.

Antagonist	Receptors	Treatment routes	Effects	Species	Ovarian states	References
Naloxone	μ- > κ- >> δ-	s.c.	Stimulatory	Rat	Ovary-intact	Ieiri et al., 1980
		i.v.	Stimulatory	Sheep	Ovary-intact	Ebling et al., 1989
		i.v.	Stimulatory	Sheep	OVX + E2	Ebling et al., 1989
		i.v.	Stimulatory	Sheep	OVX	Ebling et al., 1989
		i.v.	Stimulatory	Sheep	OVX + E2	Wood et al., 1992
nor-BNI	κ-	i.p.	Stimulatory	Rat	Ovary-intact	Nakahara et al., 2013
		i.v.	Stimulatory	Sheep	OVX + E2	Lopez et al., 2016

It has also been established that puberty onset is associated with body growth in mammals. Indeed, growth retardation resulted in delayed puberty onset in rats and sheep (Foster and Olster, 1985; Bronson, 1986; Majarune et al., 2019). Our previous study showed that chronic food restriction (negative energy balance) during the pre-pubertal phase caused suppression of ARC *Pdyn* and *Kiss1* expression and subsequent pubertal failure in growth-retarded female rats and that *ad libitum* feeding (positive energy cues) caused an acute increase in the number of *Pdyn*- and *Kiss1*-expressing cells in the ARC, triggering puberty onset in growth-retarded female rats (Majarune et al., 2019). Similarly, Aerts et al. (2021) showed pubertal increases in *Pdyn* and *Kiss1*, but not *Tac3*, expression in the ARC of lambs. These findings suggest that dynorphin-KOR signaling and *Kiss1* (as components of KNDy neurons) serve as critical regulators of GnRH pulse generation at the onset of puberty in female mammals. The completion of KNDy mRNA and peptide expression at puberty onset is likely a prerequisite. On the other hand, there might be species differences in pubertal changes in KNDy mRNA and peptide expression: Harlow et al. (2022) demonstrated that OVX lambs with all three KNDy mRNA and peptide expression showed apparent LH pulses, whereas OVX lambs under food restriction showed suppression of *Kiss1*/kisspeptin and NKB, but not *Tac3* and *Pdyn*/dynorphin, expression and the suppression of LH pulses. It should also be noted that our and other previous studies showed that ARC *Kiss1* expression was found even in neonates and did not alter peripubertal female pigs (Ieda et al., 2014; Harlow et al., 2021). Interestingly, Ebling et al. (1990) showed that IV administration of naloxone failed to affect pre-pubertal restraints of LH secretion in growth-retarded OVX lambs. Given that endogenous opioid peptides may mediate estrogen-dependent pre-pubertal suppression of GnRH/LH pulses in lambs, this finding suggests that inhibitory input(s), other than endogenous opioid peptides, may mainly mediate such steroid-independent inhibition of GnRH/LH secretion in pre-pubertal lambs under chronic malnutrition conditions.

### Candidate populations of opioidergic neurons inhibiting tonic gonadotropin-releasing hormone/gonadotropin release

It is likely that dynorphin neurons in multiple hypothalamic nuclei—such as POA, anterior hypothalamus (AHA), and PVN—and β-endorphin neurons (also known as proopiomelanocortin neurons) located in the ARC serve as mediators of the inhibitory effect of ovarian steroids and/or stressors on GnRH/gonadotropin release in female mammals.

Foradori et al. (2005) showed that ovariectomy decreased the number of *Pdyn*-expressing neurons in the POA, AHA, and ARC compared to that in ewes at the luteal phase of the estrous cycle. The study also showed that P4 replacement restored the number of *Pdyn*-expressing cells in the POA and AHA, but not the ARC, to the level noticed in ewes at the luteal phase (Foradori et al., 2005). Our recent study showed that a systemic E2 implant that mimicked the diestrous stage significantly increased *Pdyn*-expressing cells in the PVN of OVX rats compared to OVX rats without E2 replacement (Tsuchida et al., 2020). Such a stimulatory effect of E2 on *Pdyn* mRNA expression was not found in the ARC and SON, in which dynorphin neurons were also abundantly found in female rats (Kanaya et al., 2017; Tsuchida et al., 2020). Taken together, these results suggest that POA, AHA, and/or PVN dynorphin neurons may mediate the negative feedback action of ovarian steroids on pulsatile GnRH/gonadotropin release in female mammals. It is likely that P4 directly activates *Pdyn* mRNA expression in the POA and AHA because previous studies using *in situ* hybridization or immunohistochemistry revealed that the majority of dynorphin neurons in the POA and AHA expressed nuclear progesterone receptors (PR) in ewes (Foradori et al., 2002). In addition, the majority of ARC dynorphin (KNDy) neurons expressed PR and estrogen receptor α (ERα) in ewes (Foradori et al., 2002; Franceschini et al., 2006; Smith et al., 2007) and ERα in rodents (Kinoshita et al., 2005; Smith et al., 2005; Adachi et al., 2007). It is still unclear whether PVN dynorphin neurons express ERα in rats.

Palkovits (2000) demonstrated that several stressors, such as immobilization and formalin injection, induced *Pdyn* expression in the PVN, and immobilization stress induced dynorphin-immunoreactivity in the SON of female rats. Our previous study showed that glucoprivation induced by central and peripheral injection of 2DG increased the number of activated (*fos*-positive) dynorphin neurons in the PVN in OVX rats treated with a diestrous level of E2 (Tsuchida et al., 2020). Thus, it might be possible that PVN and/or SON dynorphin neurons likely mediate the suppression of pulsatile GnRH/gonadotropin release induced by stress or malnutrition in female mammals.

Interestingly, both fasting and glucoprivation suppressed LH pulses in female rats in an estrogen-dependent manner (Cagampang et al., 1991; Nagatani et al., 1996). Our previous studies showed that 48-h fasting induced *de novo* ERα expression in the PVN (Estacio et al., 1996) and that the local E2 implant into the PVN is needed for the fasting-induced suppression of LH in OVX rats (Nagatani et al., 1994). Thus, it is tempting to speculate that PVN dynorphin neurons may integrate ovarian steroid-negative feedback and stressor-induced signals to suppress GnRH/gonadotropin pulses, although the detailed phenotype of PVN ERα-expressing cells is currently unknown.

Whisnant et al. (1992) and Broad et al. (1993) showed that both E2 and P4 increased *Pomc* mRNA levels in the ARC of OVX ewes, suggesting that ARC β-endorphin neurons may mediate the negative feedback action of ovarian steroids on pulsatile GnRH/gonadotropin release at least in sheep. On the other hand, Wilcox and Roberts (1985) showed that E2 decreased *Pomc* mRNA levels in the ARC of OVX rats, indicating that there is a potential species difference in the regulation of *Pomc* mRNA expression by ovarian steroids. Little is known about stress-induced *Pomc* mRNA upregulation in female rodents, while fasting increased β-endorphin release from the hypothalamic explant of male rats (Mitev et al., 1993). Thus, further studies are needed to clarify how ARC β-endorphin neurons mediate the inhibitory effect of ovarian steroids and/or stressors on tonic GnRH/gonadotropin release in female rodents.

### Possible action sites of endogenous opioid peptides to inhibit tonic gonadotropin-releasing hormone/gonadotropin release

Receptors for endogenous opioid peptides are widely distributed in the brain of rodents (Mansour et al., 1988, 1993, 1994, 1995a; Desjardins et al., 1990; George et al., 1994). The receptor distribution was initially examined by autoradiography (at the brain nucleus level) and later examined by *in situ* hybridization (at the cell body level) and immunohistochemistry (at the cell body and fiber levels) (Mansour et al., 1995a). The difference between the localization of binding sites (detected by autoradiography) and mRNA expression (detected by *in situ* hybridization) could be explained by receptor transportation from the cell bodies to the axon terminals. It was reported that the MOR and KOR are widely distributed throughout the hypothalamus, whereas the DOR is scarcely distributed in the hypothalamus in rodents (Mansour et al., 1993, 1994, 1995a; George et al., 1994). Importantly, the distribution of opioid receptors are largely consistent between rodents and humans: the MOR and KOR mRNA are widely expressed, and DOR mRNA is rarely expressed in the human hypothalamus (Peckys and Landwehrmeyer, 1999).

**FIGURE 3:**
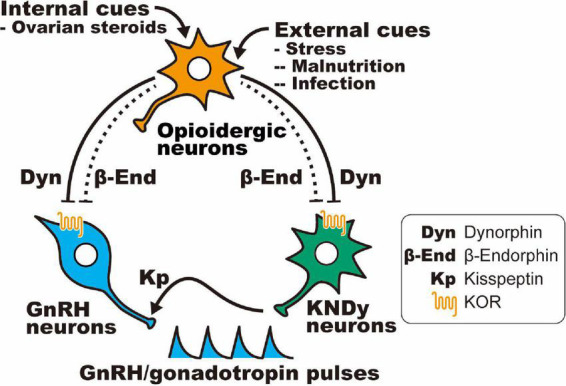
Schematic illustration showing a current interpretation of the opioidergic pathway in the regulation of GnRH/gonadotropin release in mammals: hypothalamic dynorphin and β-endorphin neurons serve as mediators of the inhibitory effect of ovarian steroids (internal cues) and/or stressors (e.g., malnutrition and infection; external cues) on GnRH/gonadotropin release in female mammals. It is likely that dynorphin directly acts on the majority of KNDy and GnRH neurons *via* the KOR, whereas β-endorphin indirectly (dotted line) acts on KNDy and GnRH neurons *via* μ-opioid receptor (MOR)-expressing interneurons.

Table 6 shows opioid receptor expression in GnRH neurons and KNDy neurons, which are considered a core component of the GnRH pulse generator, in female mammals (Lehman et al., 2010a; Okamura et al., 2013; Uenoyama et al., 2014, 2021b; Goodman et al., 2018; Moore et al., 2018; Ikegami et al., 2021). Immunohistochemical analysis revealed that the KOR is expressed in a large majority of ovine KNDy neurons and ovine and rat GnRH neurons (Lopez et al., 2016; Weems et al., 2016). In addition, *in situ* hybridization analyses revealed that KOR mRNA is expressed in the majority of ARC kisspeptin neurons in female rats (Tsuchida et al., 2020) and less than half of the ARC kisspeptin neurons in female mice (Navarro et al., 2009). Likewise, our quantitative RT-PCR analysis showed that KOR mRNA expression was detected in two of six pools of KNDy neurons (each pool consists of 10 green fluorescent protein-labeled kisspeptin cells) in female mice (Ikegami et al., 2017). On the other hand, previous *in situ* hybridization analyses revealed little MOR mRNA expression in both KNDy and GnRH neurons in female rats (Mitchell et al., 1997; Sannella and Petersen, 1997; Tsuchida et al., 2021), while MOR mRNA expression was observed in one-third of GnRH-immunoreactive cells in female guinea pigs (Zheng et al., 2005). MOR mRNA expression was found in a number of ARC non-KNDy and POA non-GnRH neurons in female rats (Mitchell et al., 1997; Sannella and Petersen, 1997; Tsuchida et al., 2021). Taken together, these findings suggest that dynorphin-KOR signaling in the majority of KNDy and GnRH neurons may mediate the negative feedback action of ovarian steroids and stress-induced suppression of tonic GnRH/gonadotropin release in female mammals (Figure 3). In addition, inhibitory β-endorphin-MOR signaling on interneurons may somehow transmit to KNDy and GnRH neurons to suppress tonic GnRH/gonadotropin release (Figure 3). To date, studies on DOR expression in KNDy or GnRH neurons are limited, while a previous study reported no DOR mRNA expression in GnRH neurons in female rats under various steroid milieus (Sannella and Petersen, 1997).

**TABLE 6:** Expression of opioid receptor mRNAs in gonadotropin-releasing hormone (GnRH) and KNDy neurons in female mammals.

Neurons	Receptors	Expression rates	Species	Gonadal states	Methods	References
GnRH	μ-	0%	Rat	Proestrus	ISH	Mitchell et al., 1997
	μ-	0%	Rat	Intact/OVX/OVX + E2/OVX + E2 + P4	ISH	Sannella and Petersen, 1997
	μ-	33.3%	Guinea pig	OVX	ISH + IHC^1^	Zheng et al., 2005
	μ-	0%	Rat	OVX + E2	ISH	Tsuchida et al., 2021
	δ-	0%	Rat	Intact/OVX/OVX + E2/OVX + E2 + P4	ISH	Sannella and Petersen, 1997
	κ-	0%	Rat	Proestrus	ISH	Mitchell et al., 1997
	κ-	0%	Rat	Intact/OVX/OVX + E2/OVX + E2 + P4	ISH	Sannella and Petersen, 1997
	κ-	95.4%	Sheep	luteal phase	IHC	Weems et al., 2016
	κ-	95.4%	Rat	OVX + E2 + P4	IHC	Weems et al., 2016
KNDy	μ-	0.4%	Rat	OVX + low E2	ISH	Tsuchida et al., 2021
	κ-	20%	Mouse	OVX/OVX + E2	ISH	Navarro et al., 2009
	κ-	33%	Mouse	OVX	qRT-PCR^2^	Ikegami et al., 2017
	κ-	97.8%	Sheep	luteal phase	IHC	Weems et al., 2016
	κ-	62%	Rat	OVX + low E2	ISH	Tsuchida et al., 2020

ISH, in situ hybridization; IHC, immunohistochemistry.

^1^GnRH neurons were detected by IHC.

^2^Oprk1 (coding KOR) expression in pooled KNDy cells was analyzed by qRT–PCR.

## Possible involvement of endogenous opioid peptides in gonadotropin-releasing hormone/luteinizing hormone surge generation

Previous studies suggest that a transient decrease in the endogenous opioid tone contributes to the initiation of the preovulatory LH surge in female mammals (Gabriel et al., 1983; Allen and Kalra, 1986; Rossmanith et al., 1988; Walsh and Clarke, 1996; Smith and Gallo, 1997). Concretely, IV or SC administration of naloxone advanced the onset of LH surge induction and increased the amplitude of LH surge in women with normal cycles (Rossmanith et al., 1988) and in proestrous or estradiol benzoate (EB)-treated OVX rats (Gabriel et al., 1983; Allen and Kalra, 1986). Furthermore, Smith and Gallo (1997) showed that nor-BNI infusion into the medial POA advanced the onset of LH surge in proestrous female rats. In addition, Walsh and Clarke (1996) showed that an MOR agonist, but not KOR and DOR agonists, delayed the onset of the EB-induced LH surge in OVX ewes. These findings suggest that endogenous opioid peptides may exert an inhibitory influence on GnRH/LH surge generation.

It is well accepted that another population of hypothalamic kisspeptin neurons, which are located in the anteroventral periventricular nucleus (AVPV) in rodents (Smith et al., 2005, 2006; Adachi et al., 2007; Clarkson et al., 2008) and the POA in several mammalian species, including macaque monkeys (Smith et al., 2010; Watanabe et al., 2014), sheep (Smith et al., 2009), goats (Matsuda et al., 2015), cattle (Hassaneen et al., 2016), and musk shrews (Inoue et al., 2011), as well as in the periventricular nucleus in pigs (Tomikawa et al., 2010), have been considered to serve as a target of estrogen-positive feedback action to induce GnRH surge in female mammals (see review article for details, Uenoyama et al., 2021a; Goodman et al., 2022; Tsukamura, 2022). Interestingly, previous studies showed coexpression of *Penk*/Met-enkephalin and *Pdyn* in the majority of AVPV kisspeptin neurons in female mice (Porteous et al., 2011; Stephens and Kauffman, 2021). To the best of our knowledge, little is known about the physiological roles of Met-enkephalin and dynorphin in AVPV kisspeptin neurons, although these findings tempt us to speculate that Met-enkephalin and/or dynorphin may have a role as a regulatory signal for LH surge generation in an autocrine/paracrine fashion in mice. Stephens and Kauffman (2021) showed that *Pdyn* expression was higher in OVX mice than E2-treated OVX mice, suggesting that dynorphin may suppress the onset of LH surge in an autocrine/paracrine fashion. Further studies are needed to uncover the precise mechanism by which endogenous opioid peptides regulate LH surge generation in female mammals.

## Conclusion and perspectives

Based on the findings currently available, we can envisage that hypothalamic opioidergic neurons play several important roles in the brain mechanism, regulating reproduction in female mammals. In particular, ARC dynorphin neurons, which are now known as KNDy neurons because of the coexpression of NKB and kisspeptin, are recognized as the GnRH pulse generator that governs female reproduction by controlling tonic GnRH/gonadotropin release throughout the estrus/menstrual cycles. In addition, dynorphin neurons located in several hypothalamic nuclei, such as the POA, AHA, and/or PVN, are likely to serve as mediators of ovarian steroid-negative feedback action on tonic GnRH/gonadotropin release by suppressing KNDy and/or GnRH neuronal activity *via* the KOR expressed in KNDy and/or GnRH neurons in female mammals. It is also postulated that ARC β-endorphin neurons may also mediate ovarian steroid-negative feedback action and suppress KNDy and GnRH neuronal activity *via* MOR-positive interneurons. Furthermore, hypothalamic opioidergic neurons are also likely to serve as mediators of external adverse cues, such as malnutrition and infection, and suppress tonic GnRH/gonadotropin release under stressful conditions. To date, findings have mainly been accumulated for MOR and KOR signaling, and little is known about whether DOR signaling serves as a mediator of ovarian steroid-negative feedback action and/or stress-induced suppression of tonic GnRH/gonadotropin release in female mammals. To uncover the precise roles of hypothalamic opioidergic neurons in mammalian reproduction as a whole, further studies are needed to clarify precise opioidergic neural pathways that control KNDy and GnRH neuronal activity in female mammals.

## Author contributions

YU and HTk collected the information and wrote the manuscript. HTc, MN, and NI critically revised the manuscript. All authors contributed to the article and approved the submitted version.

## References

[B1] Adachi S. Yamada S. Takatsu Y. Matsui H. Kinoshita M. Takase K. (2007). Involvement of anteroventral periventricular metastin/kisspeptin neurons in estrogen positive feedback action on luteinizing hormone release in female rats. *J. Reprod. Dev.* 53 367–378. 17213691 10.1262/jrd.18146

[B2] Aerts E. G. Harlow K. Griesgraber M. J. Bowdridge E. C. Hardy S. L. Nestor C. C. (2021). Kisspeptin, neurokinin B, and dynorphin expression during pubertal development in female sheep. *Biology* 10:988. 10.3390/biology10100988 34681086 PMC8533601

[B3] Akil H. Watson S. J. Young E. Lewis M. E. Khachaturian H. Walker J. M. (1984). Endogenous opioids: Biology and function. *Annu. Rev. Neurosci.* 7 223–255. 10.1146/annurev.ne.07.030184.001255 6324644

[B4] Allen L. G. Kalra S. P. (1986). Evidence that a decrease in opioid tone may evoke preovulatory luteinizing hormone release in the rat. *Endocrinology* 118 2375–2381. 10.1210/endo-118-6-2375 3698918

[B5] Amstalden M. Coolen L. M. Hemmerle A. M. Billings H. J. Connors J. M. Goodman R. L. (2010). Neurokinin 3 receptor immunoreactivity in the septal region, preoptic area and hypothalamus of the female sheep: Colocalisation in neurokinin B cells of the arcuate nucleus but not in gonadotrophin-releasing hormone neurones. *J. Neuroendocrinol.* 22 1–12. 10.1111/j.1365-2826.2009.01930.x 19912479 PMC2821793

[B6] Bale T. L. Vale W. W. (2004). CRF and CRF receptors: Role in stress responsivity and other behaviors. *Annu. Rev. Pharmacol. Toxicol.* 44 525–557. 10.1146/annurev.pharmtox.44.101802.121410 14744257

[B7] Belchetz P. E. Plant T. M. Nakai Y. Keogh E. J. Knobil E. (1978). Hypophysial responses to continuous and intermittent delivery of hypothalamic gonadotropin-releasing hormone. *Science* 202 631–633. 100883 10.1126/science.100883

[B8] Benarroch E. E. (2012). Endogenous opioid systems: Current concepts and clinical correlations. *Neurology* 79 807–814. 10.1212/WNL.0b013e3182662098 22915176

[B9] Birdsall N. J. Hulme E. C. (1976). C fragment of lipotropin has a high affinity for brain opiate receptors. *Nature* 260 793–795. 10.1038/260793a0 1264258

[B10] Bowe J. E. Li X. F. Kinsey-Jones J. S. Paterson S. Brain S. D. Lightman S. L. (2005). Calcitonin gene-related peptide-induced suppression of luteinizing hormone pulses in the rat: The role of endogenous opioid peptides. *J. Physiol.* 566 921–928. 10.1113/jphysiol.2005.085662 15905218 PMC1464796

[B11] Broad K. D. Kendrick K. M. Sirinathsinghji D. J. Keverne E. B. (1993). Changes in pro-opiomelanocortin and pre-proenkephalin mRNA levels in the ovine brain during pregnancy, parturition and lactation and in response to oestrogen and progesterone. *J. Neuroendocrinol.* 5 711–719. 10.1111/j.1365-2826.1993.tb00544.x 8680446

[B12] Bronson F. H. (1986). Food-restricted, prepubertal, female rats: Rapid recovery of luteinizing hormone pulsing with excess food, and full recovery of pubertal development with gonadotropin-releasing hormone. *Endocrinology* 118 2483–2487. 10.1210/endo-118-6-2483 3516663

[B13] Brooks A. N. Challis J. R. (1989). Effects of CRF, AVP and opioid peptides on pituitary-adrenal responses in sheep. *Peptides* 10 1291–1293. 10.1016/0196-9781(89)90024-7 2560179

[B14] Brooks A. N. Lamming G. E. Lees P. D. Haynes N. B. (1986). Opioid modulation of LH secretion in the ewe. *J. Reprod. Fertil.* 76 693–708. 10.1530/jrf.0.0760693 3701707

[B15] Brownstein M. J. (1993). A brief history of opiates, opioid peptides, and opioid receptors. *Proc. Natl. Acad. Sci. U. S. A.* 90 5391–5393. 10.1073/pnas.90.12.5391 8390660 PMC46725

[B16] Cagampang F. R. Maeda K.-I. Tsukamura H. Ohkura S. Ota K. (1991). Involvement of ovarian steroids and endogenous opioids in the fasting-induced suppression of pulsatile LH release in ovariectomized rats. *J. Endocrinol.* 129 321–328. 10.1677/joe.0.1290321 2066689

[B17] Chatterton R. T. (1990). The role of stress in female reproduction: Animal and human considerations. *Int. J. Fertil.* 35 8–13. 1968447

[B18] Chen Y. Mestek A. Liu J. Hurley J. A. Yu L. (1993a). Molecular cloning and functional expression of a μ-opioid receptor from rat brain. *Mol. Pharmacol.* 44 8–12. 8393525

[B19] Chen Y. Mestek A. Liu J. Yu L. (1993b). Molecular cloning of a rat κ opioid receptor reveals sequence similarities to the μ and δ opioid receptors. *Biochem. J.* 295 625–628. 10.1042/bj2950625 8240267 PMC1134603

[B20] Clarke I. J. Cummins J. T. (1982). The temporal relationship between gonadotropin releasing hormone (GnRH) and luteinizing hormone (LH) secretion in ovariectomized ewes. *Endocrinology* 111 1737–1739. 10.1210/endo-111-5-1737 6751801

[B21] Clarke I. J. Horton R. J. Doughton B. W. (1990). Investigation of the mechanism by which insulin-induced hypoglycemia decreases luteinizing hormone secretion in ovariectomized ewes. *Endocrinology* 127 1470–1476. 10.1210/endo-127-3-1470 2201538

[B22] Clarkson J. d’Anglemont, de Tassigny X. Moreno A. S. Colledge W. H. Herbison A. E. (2008). Kisspeptin-GPR54 signaling is essential for preovulatory gonadotropin-releasing hormone neuron activation and the luteinizing hormone surge. *J. Neurosci.* 28 8691–8697. 10.1523/JNEUROSCI.1775-08.2008 18753370 PMC6670827

[B23] Connor M. Christie M. D. (1999). Opioid receptor signalling mechanisms. *Clin. Exp. Pharmacol. Physiol.* 26 493–499. 10.1046/j.1440-1681.1999.03049.x 10405772

[B24] Desjardins G. C. Brawer J. R. Beaudet A. (1990). Distribution of mu, delta, and kappa opioid receptors in the hypothalamus of the rat. *Brain Res.* 536 114–123. 10.1016/0006-8993(90)90015-4 1964829

[B25] Devereaux A. L. Mercer S. L. Cunningham C. W. (2018). DARK Classics in Chemical Neuroscience: Morphine. *ACS Chem. Neurosci.* 9 2395–2407. 10.1021/acschemneuro.8b00150 29757600

[B26] Dyer R. G. Mansfield S. Corbet H. Dean A. D. (1985). Fasting impairs LH secretion in female rats by activating an inhibitory opioid pathway. *J. Endocrinol.* 105 91–97. 10.1677/joe.0.1050091 4039350

[B27] Ebling F. J. Schwartz M. L. Foster D. L. (1989). Endogenous opioid regulation of pulsatile luteinizing hormone secretion during sexual maturation in the female sheep. *Endocrinology* 125 369–383. 10.1210/endo-125-1-369 2737153

[B28] Ebling F. J. Wood R. I. Karsch F. J. Vannerson L. A. Suttie J. M. Bucholtz D. C. (1990). Metabolic interfaces between growth and reproduction. III. Central mechanisms controlling pulsatile luteinizing hormone secretion in the nutritionally growth-limited female lamb. *Endocrinology* 126 2719–2727. 10.1210/endo-126-5-2719 2184021

[B29] Estacio M. A. Yamada S. Tsukamura H. Hirunagi K. Maeda K.-I. (1996). Effect of fasting and immobilization stress on estrogen receptor immunoreactivity in the brain in ovariectomized female rats. *Brain Res.* 717 55–61. 8738253 10.1016/0006-8993(96)00022-4

[B30] Evans C. J. Keith D. E. Jr. Morrison H. Magendzo K. Edwards R. H. (1992). Cloning of a delta opioid receptor by functional expression. *Science* 258 1952–1955. 10.1126/science.1335167 1335167

[B31] Foradori C. D. Amstalden M. Goodman R. L. Lehman M. N. (2006). Colocalisation of dynorphin A and neurokinin B immunoreactivity in the arcuate nucleus and median eminence of the sheep. *J. Neuroendocrinol.* 18 534–541. 10.1111/j.1365-2826.2006.01445.x 16774502

[B32] Foradori C. D. Coolen L. M. Fitzgerald M. E. Skinner D. C. Goodman R. L. Lehman M. N. (2002). Colocalization of progesterone receptors in parvicellular dynorphin neurons of the ovine preoptic area and hypothalamus. *Endocrinology* 143 4366–4374. 10.1210/en.2002-220586 12399433

[B33] Foradori C. D. Goodman R. L. Adams V. L. Valent M. Lehman M. N. (2005). Progesterone increases dynorphin A concentrations in cerebrospinal fluid and preprodynorphin messenger ribonucleic acid levels in a subset of dynorphin neurons in the sheep. *Endocrinology* 146 1835–1842. 10.1210/en.2004-1326 15650077

[B34] Foster D. L. Olster D. H. (1985). Effect of restricted nutrition on puberty in the lamb: Patterns of tonic luteinizing hormone (LH) secretion and competency of the LH surge system. *Endocrinology* 116 375–381. 10.1210/endo-116-1-375 3964750

[B35] Foster D. L. Ryan K. D. (1979). Mechanisms governing onset of ovarian cyclicity at puberty in the lamb. *Ann. de Biologie Animale Biochimie Biophysique* 19 1369–1380.

[B36] Franceschini I. Lomet D. Cateau M. Delsol G. Tillet Y. Caraty A. (2006). Kisspeptin immunoreactive cells of the ovine preoptic area and arcuate nucleus co-express estrogen receptor alpha. *Neurosci. Lett.* 401 225–230. 10.1016/j.neulet.2006.03.039 16621281

[B37] Froehlich J. C. (1997). Opioid peptides. *Alcohol. Health Res. World* 21 132–136. 15704349 PMC6826828

[B38] Fukuda K. Kato S. Mori K. Nishi M. Takeshima H. (1993). Primary structures and expression from cDNAs of rat opioid receptor δ- and μ-subtypes. *FEBS Lett.* 327 311–314. 10.1016/0014-5793(93)81011-n 8394245

[B39] Gabriel S. M. Simpkins J. W. Kalra S. P. (1983). Modulation of endogenous opioid influence on luteinizing hormone secretion by progesterone and estrogen. *Endocrinology* 113 1806–1811. 10.1210/endo-113-5-1806 6313330

[B40] Gallo R. V. (1990). Kappa-opioid receptor involvement in the regulation of pulsatile luteinizing hormone release during early pregnancy in the rat. *J. Neuroendocrinol.* 2 685–691. 10.1111/j.1365-2826.1990.tb00465.x 19215406

[B41] George S. R. Zastawny R. L. Briones-Urbina R. Cheng R. Nguyen T. Heiber M. (1994). Distinct distributions of mu, delta and kappa opioid receptor mRNA in rat brain. *Biochem. Biophys. Res. Commun.* 205 1438–1444. 10.1006/bbrc.1994.2826 7802680

[B42] Gindoff P. R. Ferin M. (1987). Endogenous opioid peptides modulate the effect of corticotropin-releasing factor on gonadotropin release in the primate. *Endocrinology* 121 837–842. 10.1210/endo-121-3-837 3113919

[B43] Goldstein A. Fischli W. Lowney L. I. Hunkapiller M. Hood L. (1981). Porcine pituitary dynorphin: Complete amino acid sequence of the biologically active heptadecapeptide. *Proc. Natl. Acad. Sci. U. S. A.* 78 7219–7223. 10.1073/pnas.78.11.7219 6118870 PMC349228

[B44] Goldstein A. Tachibana S. Lowney L. I. Hunkapiller M. Hood L. (1979). Dynorphin-(1-13), an extraordinarily potent opioid peptide. *Proc. Natl. Acad. Sci. U. S. A.* 76 6666–6670. 10.1073/pnas.76.12.6666 230519 PMC411929

[B45] Goodman R. L. Coolen L. M. Anderson G. M. Hardy S. L. Valent M. Connors J. M. (2004). Evidence that dynorphin plays a major role in mediating progesterone negative feedback on gonadotropin-releasing hormone neurons in sheep. *Endocrinology* 145 2959–2967. 10.1210/en.2003-1305 14988383

[B46] Goodman R. L. Herbison A. E. Lehman M. N. Navarro V. M. (2022). Neuroendocrine control of gonadotropin-releasing hormone: Pulsatile and surge modes of secretion. *J. Neuroendocrinol.* 34:e13094. 10.1111/jne.13094 35107859 PMC9948945

[B47] Goodman R. L. Hileman S. M. Nestor C. C. Porter K. L. Connors J. M. Hardy S. L. (2013). Kisspeptin, neurokinin B, and dynorphin act in the arcuate nucleus to control activity of the GnRH pulse generator in ewes. *Endocrinology* 154 4259–4269. 10.1210/en.2013-1331 23959940 PMC3800763

[B48] Goodman R. L. Lehman M. N. Smith J. T. Coolen L. M. de Oliveira C. V. Jafarzadehshirazi M. R. (2007). Kisspeptin neurons in the arcuate nucleus of the ewe express both dynorphin A and neurokinin B. *Endocrinology* 148 5752–5760. 10.1210/en.2007-0961 17823266

[B49] Goodman R. L. Ohkura S. Okamura H. Coolen L. M. Lehman M. N. (2018). “KNDy hypothesis for generation of GnRH pulses: Evidence from sheep and goats,” in *The GnRH neuron and its control*, eds Herbison A. E. Plant T. M. (Hoboken, NJ: Wiley), 289–324.

[B50] Gottsch M. L. Cunningham M. J. Smith J. T. Popa S. M. Acohido B. V. Crowley W. F. (2004). A role for kisspeptins in the regulation of gonadotropin secretion in the mouse. *Endocrinology* 145 4073–4077. 15217982 10.1210/en.2004-0431

[B51] Gruber S. A. Silveri M. M. Yurgelun-Todd D. A. (2007). Neuropsychological consequences of opiate use. *Neuropsychol. Rev.* 17 299–315. 10.1007/s11065-007-9041-y 17690984

[B52] Harlow K. Griesgraber M. J. Seman A. D. Shuping S. L. Sommer J. R. Griffith E. H. (2022). The impact of undernutrition on KNDy (kisspeptin/neurokinin B/dynorphin) neurons in female lambs. *J. Neuroendocrinol.* 34:e13135. 10.1111/jne.13135 35579068 PMC9286635

[B53] Harlow K. Renwick A. N. Shuping S. L. Sommer J. R. Lents C. A. Knauer M. T. (2021). Evidence that pubertal status impacts kisspeptin/neurokinin B/dynorphin neurons in the giltdagger. *Biol. Reprod.* 105 1533–1544. 10.1093/biolre/ioab189 34643223

[B54] Hassaneen A. S. A. Naniwa Y. Suetomi Y. Matsuyama S. Kimura K. Ieda N. (2016). Immunohistochemical characterization of the arcuate kisspeptin/neurokinin B/dynorphin (KNDy) and preoptic kisspeptin neuronal populations in the hypothalamus during the estrous cycle in heifers. *J. Reprod. Dev.* 62 471–477. 10.1262/jrd.2016-075 27349533 PMC5081734

[B55] Herbison A. E. (2020). A simple model of estrous cycle negative and positive feedback regulation of GnRH secretion. *Front. Neuroendocrinol.* 57:100837. 10.1016/j.yfrne.2020.100837 32240664

[B56] Herbison A. E. de Tassigny X. Doran J. Colledge W. H. (2010). Distribution and postnatal development of Gpr54 gene expression in mouse brain and gonadotropin-releasing hormone neurons. *Endocrinology* 151 312–321. 10.1210/en.2009-0552 19966188

[B57] Higo S. Honda S. Iijima N. Ozawa H. (2016). Mapping of kisspeptin receptor mRNA in the whole rat brain and its co-localisation with oxytocin in the paraventricular nucleus. *J. Neuroendocrinol.* 28 10.1111/jne.12356 26709462

[B58] Hrabovszky E. Sipos M. T. Molnar C. S. Ciofi P. Borsay B. A. Gergely P. (2012). Low degree of overlap between kisspeptin, neurokinin B, and dynorphin immunoreactivities in the infundibular nucleus of young male human subjects challenges the KNDy neuron concept. *Endocrinology* 153 4978–4989. 10.1210/en.2012-1545 22903610 PMC3512020

[B59] Hrabovszky E. Takacs S. Gocz B. Skrapits K. (2019). New perspectives for anatomical and molecular studies of kisspeptin neurons in the aging human brain. *Neuroendocrinology* 109 230–241. 10.1159/000496566 30612127

[B60] Hughes J. Smith T. Morgan B. Fothergill L. (1975a). Purification and properties of enkephalin - the possible endogenous ligand for the morphine receptor. *Life Sci.* 16 1753–1758. 10.1016/0024-3205(75)90268-4 1152599

[B61] Hughes J. Smith T. W. Kosterlitz H. W. Fothergill L. A. Morgan B. A. Morris H. R. (1975b). Identification of two related pentapeptides from the brain with potent opiate agonist activity. *Nature* 258 577–580. 10.1038/258577a0 1207728

[B62] Hulse G. K. Coleman G. J. (1983). The role of endogenous opioids in the blockade of reproductive function in the rat following exposure to acute stress. *Pharmacol. Biochem. Behav.* 19 795–799. 10.1016/0091-3057(83)90083-7 6685880

[B63] Ieda N. Uenoyama Y. Tajima Y. Nakata T. Kano M. Naniwa Y. (2014). KISS1 gene expression in the developing brain of female pigs in pre- and peripubertal periods. *J. Reprod. Dev.* 60 312–316. 10.1262/jrd.2013-129 24909600 PMC4139506

[B64] Ieiri T. Chen H. T. Meites J. (1980). Naloxone stimulation of luteinizing hormone release in prepubertal female rats; role of serotonergic system. *Life Sci.* 26 1269–1274. 10.1016/0024-3205(80)90072-7 6446634

[B65] Ikegami K. Minabe S. Ieda N. Goto T. Sugimoto A. Nakamura S. (2017). Evidence of involvement of neurone-glia/neurone-neurone communications via gap junctions in synchronised activity of KNDy neurones. *J. Neuroendocrinol.* 29 10.1111/jne.12480 28475285

[B66] Ikegami K. Watanabe Y. Nakamura S. Goto T. Inoue N. Uenoyama Y. (2021). Cellular and molecular mechanisms regulating the KNDy neuronal activities to generate and modulate GnRH pulse in mammals. *Front. Neuroendocrinol.* 64:100968. 10.1016/j.yfrne.2021.100968 34808231

[B67] Inoue N. Sasagawa K. Ikai K. Sasaki Y. Tomikawa J. Oishi S. (2011). Kisspeptin neurons mediate reflex ovulation in the musk shrew (Suncus murinus). *Proc. Natl. Acad. Sci. U. S. A.* 108 17527–17532. 10.1073/pnas.1113035108 21987818 PMC3198342

[B68] Irwig M. S. Fraley G. S. Smith J. T. Acohido B. V. Popa S. M. Cunningham M. J. (2004). Kisspeptin activation of gonadotropin releasing hormone neurons and regulation of KiSS-1 mRNA in the male rat. *Neuroendocrinology* 80 264–272. 15665556 10.1159/000083140

[B69] Kakidani H. Furutani Y. Takahashi H. Noda M. Morimoto Y. Hirose T. (1982). Cloning and sequence analysis of cDNA for porcine beta-neo-endorphin/dynorphin precursor. *Nature* 298 245–249. 10.1038/298245a0 6123953

[B70] Kanaya M. Iwata K. Ozawa H. (2017). Distinct dynorphin expression patterns with low- and high-dose estrogen treatment in the arcuate nucleus of female rats. *Biol. Reprod.* 97 709–718. 10.1093/biolre/iox131 29069289

[B71] Kieffer B. L. Befort K. Gaveriaux-Ruff C. Hirth C. G. (1992). The δ-opioid receptor: Isolation of a cDNA by expression cloning and pharmacological characterization. *Proc. Natl. Acad. Sci. U. S. A.* 89 12048–12052. 10.1073/pnas.89.24.12048 1334555 PMC50695

[B72] Kinoshita M. Tsukamura H. Adachi S. Matsui H. Uenoyama Y. Iwata K. (2005). Involvement of central metastin in the regulation of preovulatory luteinizing hormone surge and estrous cyclicity in female rats. *Endocrinology* 146 4431–4436. 10.1210/en.2005-0195 15976058

[B73] Kujjo L. L. Bosu W. T. Perez G. I. (1995). Opioid peptides involvement in endotoxin-induced suppression of LH secretion in ovariectomized Holstein heifers. *Reprod. Toxicol.* 9 169–174. 10.1016/0890-6238(94)00068-9 7795327

[B74] Lehman M. N. Coolen L. M. Goodman R. L. (2010a). Minireview: Kisspeptin/neurokinin B/dynorphin (KNDy) cells of the arcuate nucleus: A central node in the control of gonadotropin-releasing hormone secretion. *Endocrinology* 151 3479–3489. 10.1210/en.2010-0022 20501670 PMC2940527

[B75] Lehman M. N. Merkley C. M. Coolen L. M. Goodman R. L. (2010b). Anatomy of the kisspeptin neural network in mammals. *Brain Res.* 1364 90–102. 10.1016/j.brainres.2010.09.020 20858464 PMC2992597

[B76] Lehman M. N. Robinson J. E. Karsch F. J. Silverman A. J. (1986). Immunocytochemical localization of luteinizing hormone-releasing hormone (LHRH) pathways in the sheep brain during anestrus and the mid-luteal phase of the estrous cycle. *J. Comp. Neurol.* 244 19–35. 10.1002/cne.902440103 3512631

[B77] Li C. H. Chung D. (1976). Isolation and structure of an untriakontapeptide with opiate activity from camel pituitary glands. *Proc. Natl. Acad. Sci. U. S. A.* 73 1145–1148. 10.1073/pnas.73.4.1145 1063395 PMC430217

[B78] Li S. Zhu J. Chen C. Chen Y. W. Deriel J. K. Ashby B. (1993). Molecular cloning and expression of a rat κ opioid receptor. *Biochem. J.* 295 629–633. 10.1042/bj2950629 8240268 PMC1134604

[B79] Li X. F. Bowe J. E. Mitchell J. C. Brain S. D. Lightman S. L. O’Byrne K. T. (2004). Stress-induced suppression of the gonadotropin-releasing hormone pulse generator in the female rat: A novel neural action for calcitonin gene-related peptide. *Endocrinology* 145 1556–1563. 10.1210/en.2003-1609 14736738

[B80] Lincoln D. W. Fraser H. M. Lincoln G. A. Martin G. B. McNeilly A. S. (1985). Hypothalamic pulse generators. *Recent Prog. Horm. Res.* 41 369–419. 3901163 10.1016/b978-0-12-571141-8.50013-5

[B81] Lopez J. A. Bedenbaugh M. N. McCosh R. B. Weems P. W. Meadows L. J. Wisman B. (2016). Does dynorphin play a role in the onset of puberty in female sheep?. *J. Neuroendocrinol.* 28 10.1111/jne.12445 28328155 PMC5412962

[B82] Maeda K.-I. Ohkura S. Uenoyama Y. Wakabayashi Y. Oka Y. Tsukamura H. (2010). Neurobiological mechanisms underlying GnRH pulse generation by the hypothalamus. *Brain Res.* 1364 103–115. 10.1016/j.brainres.2010.10.026 20951683

[B83] Maeda K.-I. Tsukamura H. Ohkura S. Kawakami S. Nagabukuro H. Yokoyama A. (1995). The LHRH pulse generator: A mediobasal hypothalamic location. *Neurosci. Biobehav. Rev.* 19 427–437. 10.1016/0149-7634(94)00069-d 7566744

[B84] Majarune S. Nima P. Sugimoto A. Nagae M. Inoue N. Tsukamura H. (2019). Ad libitum feeding triggers puberty onset associated with increases in arcuate Kiss1 and Pdyn expression in growth-retarded rats. *J. Reprod. Dev.* 65 397–406. 10.1262/jrd.2019-048 31155522 PMC6815743

[B85] Malven P. V. Bossut D. F. Diekman M. A. (1984). Effects of naloxone and electroacupuncture treatment on plasma concentrations of LH in sheep. *J. Endocrinol.* 101 75–80. 10.1677/joe.0.1010075 6608571

[B86] Mansour A. Hoversten M. T. Taylor L. P. Watson S. J. Akil H. (1995b). The cloned μ, δ and κ receptors and their endogenous ligands: Evidence for two opioid peptide recognition cores. *Brain Res.* 700 89–98. 10.1016/0006-8993(95)00928-j 8624732

[B87] Mansour A. Fox C. A. Akil H. Watson S. J. (1995a). Opioid-receptor mRNA expression in the rat CNS: Anatomical and functional implications. *Trends Neurosci.* 18 22–29. 10.1016/0166-2236(95)93946-u 7535487

[B88] Mansour A. Fox C. A. Burke S. Meng F. Thompson R. C. Akil H. (1994). Mu, delta, and kappa opioid receptor mRNA expression in the rat CNS: An in situ hybridization study. *J. Comp. Neurol.* 350 412–438. 10.1002/cne.903500307 7884049

[B89] Mansour A. Khachaturian H. Lewis M. E. Akil H. Watson S. J. (1988). Anatomy of CNS opioid receptors. *Trends Neurosci.* 11 308–314. 10.1016/0166-2236(88)90093-8 2465635

[B90] Mansour A. Thompson R. C. Akil H. Watson S. J. (1993). Delta opioid receptor mRNA distribution in the brain: Comparison to delta receptor binding and proenkephalin mRNA. *J. Chem. Neuroanat.* 6 351–362. 10.1016/0891-0618(93)90010-2 8142072

[B91] Matsuda F. Nakatsukasa K. Suetomi Y. Naniwa Y. Ito D. Inoue N. (2015). The LH surge-generating system is functional in male goats as in females: Involvement of kisspeptin neurones in the medial preoptic area. *J. Neuroendocrinol.* 27 57–65. 10.1111/jne.12235 25367275

[B92] Messager S. Chatzidaki E. E. Ma D. Hendrick A. G. Zahn D. Dixon J. (2005). Kisspeptin directly stimulates gonadotropin-releasing hormone release via G protein-coupled receptor 54. *Proc. Natl. Acad. Sci. U. S. A.* 102 1761–1766. 10.1073/pnas.0409330102 15665093 PMC545088

[B93] Minami M. Toya T. Katao Y. Maekawa K. Nakamura S. Onogi T. (1993). Cloning and expression of a cDNA for the rat κ-opioid receptor. *FEBS Lett.* 329 291–295. 10.1016/0014-5793(93)80240-u 8103466

[B94] Mitchell V. Prevot V. Jennes L. Aubert J. P. Croix D. Beauvillain J. C. (1997). Presence of μ and κ opioid receptor mRNAs in galanin but not in GnRH neurons in the female rat. *Neuroreport* 8 3167–3172. 10.1097/00001756-199709290-00032 9331935

[B95] Mitev Y. Almeida O. F. Patchev V. (1993). Pituitary-adrenal function and hypothalamic beta-endorphin release in vitro following food deprivation. *Brain Res. Bull.* 30 7–10. 10.1016/0361-9230(93)90033-8 8420637

[B96] Moenter S. M. Brand R. M. Midgley A. R. Karsch F. J. (1992). Dynamics of gonadotropin-releasing hormone release during a pulse. *Endocrinology* 130 503–510. 1727719 10.1210/endo.130.1.1727719

[B97] Moore A. M. Coolen L. M. Porter D. T. Goodman R. L. Lehman M. N. (2018). KNDy Cells Revisited. *Endocrinology* 159 3219–3234. 10.1210/en.2018-00389 30010844 PMC6098225

[B98] Mostari M. P. Ieda N. Deura C. Minabe S. Yamada S. Uenoyama Y. (2013). Dynorphin-kappa opioid receptor signaling partly mediates estrogen negative feedback effect on LH pulses in female rats. *J. Reprod. Dev.* 59 266–272. 10.1262/jrd.2012-193 23391862 PMC3934128

[B99] Murakawa H. Iwata K. Takeshita T. Ozawa H. (2016). Immunoelectron microscopic observation of the subcellular localization of kisspeptin, neurokinin B and dynorphin A in KNDy neurons in the arcuate nucleus of the female rat. *Neurosci. Lett.* 612 161–166. 10.1016/j.neulet.2015.12.008 26679227

[B100] Nagae M. Uenoyama Y. Okamoto S. Tsuchida H. Ikegami K. Goto T. (2021). Direct evidence that KNDy neurons maintain gonadotropin pulses and folliculogenesis as the GnRH pulse generator. *Proc. Natl. Acad. Sci. U. S. A.* 118:e2009156118. 10.1073/pnas.2009156118 33500349 PMC7865162

[B101] Nagatani S. Bucholtz D. C. Murahashi K. Estacio M. A. Tsukamura H. Foster D. L. (1996). Reduction of glucose availability suppresses pulsatile luteinizing hormone release in female and male rats. *Endocrinology* 137 1166–1170. 10.1210/endo.137.4.8625885 8625885

[B102] Nagatani S. Tsukamura H. Maeda K.-I. (1994). Estrogen feedback needed at the paraventricular nucleus or A2 to suppress pulsatile luteinizing hormone release in fasting female rats. *Endocrinology* 135 870–875. 8070380 10.1210/endo.135.3.8070380

[B103] Nakahara T. Uenoyama Y. Iwase A. Oishi S. Nakamura S. Minabe S. (2013). Chronic peripheral administration of kappa-opioid receptor antagonist advances puberty onset associated with acceleration of pulsatile luteinizing hormone secretion in female rats. *J. Reprod. Dev.* 59 479–484. 10.1262/jrd.2013-046 23877505 PMC3934117

[B104] Nakamura S. Wakabayashi Y. Yamamura T. Ohkura S. Matsuyama S. (2017). A neurokinin 3 receptor-selective agonist accelerates pulsatile luteinizing hormone secretion in lactating cattle. *Biol. Reprod.* 97 81–90. 10.1093/biolre/iox068 28859282

[B105] Nakanishi S. Inoue A. Kita T. Nakamura M. Chang A. C. Cohen S. N. (1979). Nucleotide sequence of cloned cDNA for bovine corticotropin-beta-lipotropin precursor. *Nature* 278 423–427. 10.1038/278423a0 221818

[B106] Naniwa Y. Nakatsukasa K. Setsuda S. Oishi S. Fujii N. Matsuda F. (2013). Effects of full-length kisspeptin administration on follicular development in Japanese Black beef cows. *J. Reprod. Dev.* 59 588–594. 10.1262/jrd.2013-064 24107742 PMC3934150

[B107] Navarro V. M. Castellano J. M. McConkey S. M. Pineda R. Ruiz-Pino F. Pinilla L. (2011). Interactions between kisspeptin and neurokinin B in the control of GnRH secretion in the female rat. *Am. J. Physiol. Endocrinol. Metab.* 300 E202–E210. 10.1152/ajpendo.00517.2010 21045176 PMC3774070

[B108] Navarro V. M. Gottsch M. L. Chavkin C. Okamura H. Clifton D. K. Steiner R. A. (2009). Regulation of gonadotropin-releasing hormone secretion by kisspeptin/dynorphin/neurokinin B neurons in the arcuate nucleus of the mouse. *J. Neurosci.* 29 11859–11866. 10.1523/JNEUROSCI.1569-09.2009 19776272 PMC2793332

[B109] Nishi M. Takeshima H. Fukuda K. Kato S. Mori K. (1993). cDNA cloning and pharmacological characterization of an opioid receptor with high affinities for κ-subtype-selective ligands. *FEBS Lett.* 330 77–80. 10.1016/0014-5793(93)80923-i 8396539

[B110] Nishihara M. Takeuchi Y. Tanaka T. Mori Y. (1999). Electrophysiological correlates of pulsatile and surge gonadotrophin secretion. *Rev. Reprod.* 4 110–116. 10.1530/ror.0.0040110 10357098

[B111] Noda M. Furutani Y. Takahashi H. Toyosato M. Hirose T. Inayama S. (1982). Cloning and sequence analysis of cDNA for bovine adrenal preproenkephalin. *Nature* 295 202–206. 10.1038/295202a0 6276759

[B112] Ohkura S. Takase K. Matsuyama S. Mogi K. Ichimaru T. Wakabayashi Y. (2009). Gonadotrophin-releasing hormone pulse generator activity in the hypothalamus of the goat. *J. Neuroendocrinol.* 21 813–821. 10.1111/j.1365-2826.2009.01909.x 19678868

[B113] Okamura H. Tsukamura H. Ohkura S. Uenoyama Y. Wakabayashi Y. Maeda K.-I. (2013). “Kisspeptin and GnRH pulse generation,” in *Kisspeptin signaling in reproductive biology. advances in experimental medicine and biology*, eds Kauffman A. Smith J. (New York: Springer), 297–323. 10.1007/978-1-4614-6199-9_14 23550012

[B114] Palkovits M. (2000). Stress-induced expression of co-localized neuropeptides in hypothalamic and amygdaloid neurons. *Eur. J. Pharmacol.* 405 161–166. 10.1016/s0014-2999(00)00549-5 11033323

[B115] Papadimitriou A. Priftis K. N. (2009). Regulation of the hypothalamic-pituitary-adrenal axis. *Neuroimmunomodulation* 16 265–271. 10.1159/000216184 19571587

[B116] Peckys D. Landwehrmeyer G. B. (1999). Expression of mu, kappa, and delta opioid receptor messenger RNA in the human CNS: A 33P in situ hybridization study. *Neuroscience* 88 1093–1135. 10.1016/s0306-4522(98)00251-6 10336124

[B117] Pert C. B. Snyder S. H. (1973). Opiate receptor: Demonstration in nervous tissue. *Science* 179 1011–1014. 10.1126/science.179.4077.1011 4687585

[B118] Petraglia F. Locatelli V. Penalva A. Cocchi D. Genazzani A. R. Muller E. E. (1984). Gonadal steroid modulation of naloxone-induced LH secretion in the rat. *J. Endocrinol.* 101 33–39. 10.1677/joe.0.1010033 6368728

[B119] Pheng V. Uenoyama Y. Homma T. Inamoto Y. Takase K. Yoshizawa-Kumagaye K. (2009). Potencies of centrally- or peripherally-injected full-length kisspeptin or its C-terminal decapeptide on LH release in intact male rats. *J. Reprod. Dev.* 55 378–382. 10.1262/jrd.20240 19384054

[B120] Porteous R. Petersen S. L. Yeo S. H. Bhattarai J. P. Ciofi P. de Tassigny X. D. (2011). Kisspeptin neurons co-express met-enkephalin and galanin in the rostral periventricular region of the female mouse hypothalamus. *J. Comp. Neurol.* 519 3456–3469. 10.1002/cne.22716 21800299

[B121] Przewlocki R. (2013). “Opioid peptides,” in *Neuroscience in the 21st Century*, ed. Pfaff D. W. (New York: Springer), 1525–1553.

[B122] Quigley M. E. Yen S. S. (1980). The role of endogenous opiates in LH secretion during the menstrual cycle. *J. Clin. Endocrinol. Metab.* 51 179–181. 10.1210/jcem-51-1-179 7380991

[B123] Ramaswamy S. Seminara S. B. Ali B. Ciofi P. Amin N. A. Plant T. M. (2010). Neurokinin B stimulates GnRH release in the male monkey (*Macaca mulatta*) and is colocalized with kisspeptin in the arcuate nucleus. *Endocrinology* 151 4494–4503. 10.1210/en.2010-0223 20573725 PMC2940495

[B124] Raynor K. Kong H. Chen Y. Yasuda K. Yu L. Bell G. I. (1994). Pharmacological characterization of the cloned κ-, δ-, and μ-opioid receptors. *Mol. Pharmacol.* 45 330–334. 8114680

[B125] Reid R. L. Quigley M. E. Yen S. S. (1983). The disappearance of opioidergic regulation of gonadotropin secretion in postmenopausal women. *J. Clin. Endocrinol. Metab.* 57 1107–1110. 10.1210/jcem-57-6-1107 6313728

[B126] Rivest S. Plotsky P. M. Rivier C. (1993). CRF alters the infundibular LHRH secretory system from the medial preoptic area of female rats: Possible involvement of opioid receptors. *Neuroendocrinology* 57 236–246. 10.1159/000126365 8389996

[B127] Rossmanith W. G. Mortola J. F. Yen S. S. (1988). Role of endogenous opioid peptides in the initiation of the midcycle luteinizing hormone surge in normal cycling women. *J. Clin. Endocrinol. Metab.* 67 695–700. 10.1210/jcem-67-4-695 3138276

[B128] Ruiz-Pino F. Garcia-Galiano D. Manfredi-Lozano M. Leon S. Sanchez-Garrido M. A. Roa J. (2015). Effects and interactions of tachykinins and dynorphin on FSH and LH secretion in developing and adult rats. *Endocrinology* 156 576–588. 10.1210/en.2014-1026 25490143 PMC4298329

[B129] Sakamoto K. Murata K. Wakabayashi Y. Yayou K. I. Ohkura S. Takeuchi Y. (2012). Central administration of neurokinin B activates kisspeptin/NKB neurons in the arcuate nucleus and stimulates luteinizing hormone secretion in ewes during the non-breeding season. *J. Reprod. Dev.* 58 700–706. 10.1262/jrd.2011-038 22972185

[B130] Sannella M. I. Petersen S. L. (1997). Dual label in situ hybridization studies provide evidence that luteinizing hormone-releasing hormone neurons do not synthesize messenger ribonucleic acid for μ, κ, or δ opiate receptors. *Endocrinology* 138 1667–1672. 9075729 10.1210/endo.138.4.5091

[B131] Sasaki T. Ito D. Sonoda T. Morita Y. Wakabayashi Y. Yamamura T. (2019). Peripheral administration of κ-opioid receptor antagonist stimulates gonadotropin-releasing hormone pulse generator activity in ovariectomized, estrogen-treated female goats. *Domest. Anim. Endocrinol.* 68 83–91. 10.1016/j.domaniend.2018.12.011 30908995

[B132] Sasaki T. Sonoda T. Tatebayashi R. Kitagawa Y. Oishi S. Yamamoto K. (2020). Peripheral administration of SB223412, a selective neurokinin-3 receptor antagonist, suppresses pulsatile luteinizing hormone secretion by acting on the gonadotropin-releasing hormone pulse generator in estrogen-treated ovariectomized female goats. *J. Reprod. Dev.* 66 351–357. 10.1262/jrd.2019-145 32281549 PMC7470901

[B133] Senn M. Maier P. M. Langhans W. (1995). ACTH, cortisol and glucose responses after administration of vasopressin in cattle and sheep. *J. Comp. Physiol. B.* 164 570–578. 10.1007/BF00261398 7884067

[B134] Simon E. J. Hiller J. M. Edelman I. (1973). Stereospecific binding of the potent narcotic analgesic (3H) Etorphine to rat-brain homogenate. *Proc. Natl. Acad. Sci. U. S. A.* 70 1947–1949. 10.1073/pnas.70.7.1947 4516196 PMC433639

[B135] Smith J. T. Clay C. M. Caraty A. Clarke I. J. (2007). KiSS-1 messenger ribonucleic acid expression in the hypothalamus of the ewe is regulated by sex steroids and season. *Endocrinology* 148 1150–1157. 10.1210/en.2006-1435 17185374

[B136] Smith J. T. Cunningham M. J. Rissman E. F. Clifton D. K. Steiner R. A. (2005). Regulation of Kiss1 gene expression in the brain of the female mouse. *Endocrinology* 146 3686–3692. 10.1210/en.2005-0488 15919741

[B137] Smith J. T. Li Q. Pereira A. Clarke I. J. (2009). Kisspeptin neurons in the ovine arcuate nucleus and preoptic area are involved in the preovulatory luteinizing hormone surge. *Endocrinology* 150 5530–5538. 10.1210/en.2009-0712 19819940

[B138] Smith J. T. Popa S. M. Clifton D. K. Hoffman G. E. Steiner R. A. (2006). Kiss1 neurons in the forebrain as central processors for generating the preovulatory luteinizing hormone surge. *J. Neurosci.* 26 6687–6694. 10.1523/JNEUROSCI.1618-06.2006 16793876 PMC6673844

[B139] Smith J. T. Shahab M. Pereira A. Pau K. Y. Clarke I. J. (2010). Hypothalamic expression of KISS1 and gonadotropin inhibitory hormone genes during the menstrual cycle of a non-human primate. *Biol. Reprod.* 83 568–577. 10.1095/biolreprod.110.085407 20574054 PMC2957156

[B140] Smith M. J. Gallo R. V. (1997). The effect of blockade of kappa-opioid receptors in the medial preoptic area on the luteinizing hormone surge in the proestrous rat. *Brain Res.* 768 111–119. 10.1016/s0006-8993(97)00622-7 9369307

[B141] Snyder S. H. Pasternak G. W. (2003). Historical review: Opioid receptors. *Trends Pharmacol. Sci.* 24 198–205. 10.1016/S0165-6147(03)00066-X 12707007

[B142] Stein C. (2016). Opioid Receptors. *Annu. Rev. Med.* 67 433–451. 10.1146/annurev-med-062613-093100 26332001

[B143] Stephens S. B. Z. Kauffman A. S. (2021). Estrogen regulation of the molecular phenotype and active translatome of AVPV kisspeptin neurons. *Endocrinology* 162:bqab080. 10.1210/endocr/bqab080 33856454 PMC8286094

[B144] Tanaka T. Ohkura S. Wakabayashi K. Okamura H. (2012). Effect of peripherally administered kisspeptin-10 on GnRH neurosecretion into the hypophyseal portal circulation in ovariectomized goat does. *Small Ruminant Res.* 105 273–276.

[B145] Terenius L. (1973). Characteristics of the “receptor” for narcotic analgesics in synaptic plasma membrane fraction from rat brain. *Acta Pharmacol. Toxicol.* 33 377–384. 10.1111/j.1600-0773.1973.tb01539.x 4801083

[B146] Thompson R. C. Mansour A. Akil H. Watson S. J. (1993). Cloning and pharmacological characterization of a rat μ opioid receptor. *Neuron* 11 903–913. 10.1016/0896-6273(93)90120-g 8240812

[B147] Tilbrook A. J. Turner A. I. Clarke I. J. (2000). Effects of stress on reproduction in non-rodent mammals: The role of glucocorticoids and sex differences. *Rev. Reprod.* 5 105–113. 10.1530/ror.0.0050105 10864855

[B148] Tilbrook A. J. Turner A. I. Clarke I. J. (2002). Stress and reproduction: Central mechanisms and sex differences in non-rodent species. *Stress* 5 83–100. 10.1080/10253890290027912 12186687

[B149] Tomikawa J. Homma T. Tajima S. Shibata T. Inamoto Y. Takase K. (2010). Molecular characterization and estrogen regulation of hypothalamic KISS1 gene in the pig. *Biol. Reprod.* 82 313–319. 10.1095/biolreprod.109.079863 19828777

[B150] True C. Kirigiti M. Ciofi P. Grove K. L. Smith M. S. (2011). Characterisation of arcuate nucleus kisspeptin/neurokinin B neuronal projections and regulation during lactation in the rat. *J. Neuroendocrinol.* 23 52–64. 10.1111/j.1365-2826.2010.02076.x 21029216 PMC3118985

[B151] True C. Takahashi D. Kirigiti M. Lindsley S. R. Moctezuma C. Arik A. (2017). Arcuate nucleus neuropeptide coexpression and connections to gonadotrophin-releasing hormone neurones in the female rhesus macaque. *J. Neuroendocrinol.* 29 10.1111/jne.12491 28561903 PMC5523807

[B152] Tsuchida H. Kawai N. Yamada K. Takizawa M. Inoue N. Uenoyama Y. (2021). Central μ-opioid receptor antagonism blocks glucoprivic LH pulse suppression and gluconeogenesis/feeding in female rats. *Endocrinology* 162:bqab140. 10.1210/endocr/bqab140 34270714

[B153] Tsuchida H. Mostari P. Yamada K. Miyazaki S. Enomoto Y. Inoue N. (2020). Paraventricular dynorphin A neurons mediate LH pulse suppression induced by hindbrain glucoprivation in female rats. *Endocrinology* 161:bqaa161. 10.1210/endocr/bqaa161 32894768

[B154] Tsukamura H. (2022). Kobayashi Award 2019: The neuroendocrine regulation of the mammalian reproduction. *Gen. Comp. Endocrinol.* 315:113755. 10.1016/j.ygcen.2021.113755 33711315

[B155] Uenoyama Y. Inoue N. Nakamura S. Tsukamura H. (2019). Central mechanism controlling pubertal onset in mammals: A triggering role of kisspeptin. *Front. Endocrinol.* 10:312. 10.3389/fendo.2019.00312 31164866 PMC6536648

[B156] Uenoyama Y. Nagae M. Tsuchida H. Inoue N. Tsukamura H. (2021b). Role of KNDy neurons expressing kisspeptin, neurokinin B, and dynorphin A as a GnRH pulse generator controlling mammalian reproduction. *Front. Endocrinol.* 12:724632. 10.3389/fendo.2021.724632 34566891 PMC8458932

[B157] Uenoyama Y. Inoue N. Nakamura S. Tsukamura H. (2021a). Kisspeptin neurons and estrogen-estrogen receptor α signaling: Unraveling the mystery of steroid feedback system regulating mammalian reproduction. *Int. J. Mol. Sci.* 22:9229. 10.3390/ijms22179229 34502135 PMC8430864

[B158] Uenoyama Y. Tsukamura H. Maeda K.-I. (2014). KNDy neuron as a gatekeeper of puberty onset. *J. Obstet. Gynaecol. Res.* 40 1518–1526. 10.1111/jog.12398 24888910

[B159] Van Vugt D. A. Bakst G. Dyrenfurth I. Ferin M. (1983). Naloxone stimulation of luteinizing hormone secretion in the female monkey: Influence of endocrine and experimental conditions. *Endocrinology* 113 1858–1864. 10.1210/endo-113-5-1858 6414806

[B160] Wakabayashi Y. Nakada T. Murata K. Ohkura S. Mogi K. Navarro V. M. (2010). Neurokinin B and dynorphin A in kisspeptin neurons of the arcuate nucleus participate in generation of periodic oscillation of neural activity driving pulsatile gonadotropin-releasing hormone secretion in the goat. *J. Neurosci.* 30 3124–3132. 10.1523/JNEUROSCI.5848-09.2010 20181609 PMC6633939

[B161] Waldhoer M. Bartlett S. E. Whistler J. L. (2004). Opioid receptors. *Annu. Rev. Biochem.* 73 953–990. 10.1146/annurev.biochem.73.011303.073940 15189164

[B162] Walsh J. P. Clarke I. J. (1996). Effects of central administration of highly selective opioid mu-, delta- and kappa-receptor agonists on plasma luteinizing hormone (LH), prolactin, and the estrogen-induced LH surge in ovariectomized ewes. *Endocrinology* 137 3640–3648. 10.1210/endo.137.9.8756528 8756528

[B163] Watanabe Y. Uenoyama Y. Suzuki J. Takase K. Suetomi Y. Ohkura S. (2014). Oestrogen-induced activation of preoptic kisspeptin neurones may be involved in the luteinizing hormone surge in male and female Japanese monkeys. *J. Neuroendocrinol.* 26 909–917. 10.1111/jne.12227 25283748

[B164] Weems P. W. Witty C. F. Amstalden M. Coolen L. M. Goodman R. L. Lehman M. N. (2016). Kappa opioid receptor is co-localized in GnRH and KNDy cells in the female ovine and rat brain. *Endocrinology* 157 2367–2379. 10.1210/en.2015-1763 27064940 PMC4891780

[B165] Whisnant C. S. Curto K. Goodman R. L. (1992). Immunocytochemical localization of beta endorphin and gonadal steroid regulation of proopiomelanocortin messenger ribonucleic acid in the ewe. *Neuroendocrinology* 56 812–821. 10.1159/000126311 1369589

[B166] Whisnant C. S. Goodman R. L. (1988). Effects of an opioid antagonist on pulsatile luteinizing hormone secretion in the ewe vary with changes in steroid negative feedback. *Biol. Reprod.* 39 1032–1038. 10.1095/biolreprod39.5.1032 3219376

[B167] Wilcox J. N. Roberts J. L. (1985). Estrogen decreases rat hypothalamic proopiomelanocortin messenger ribonucleic acid levels. *Endocrinology* 117 2392–2396. 10.1210/endo-117-6-2392 2933246

[B168] Wood R. I. I Anson H. Ebling F. J. Foster D. L. (1992). Opioid inhibition of luteinizing hormone secretion compared in developing male and female sheep. *Neuroendocrinology* 56 822–830. 10.1159/000126312 1369590

[B169] Xiao E. Xia-Zhang L. Ferin M. (2000). Inhibitory effects of endotoxin on LH secretion in the ovariectomized monkey are prevented by naloxone but not by an interleukin-1 receptor antagonist. *Neuroimmunomodulation* 7 6–15. 10.1159/000026415 10601814

[B170] Xiao E. Xia-Zhang L. Thornell D. Shalts E. Ferin M. (1996). The luteinizing hormone but not the cortisol response to arginine vasopressin is prevented by naloxone and a corticotropin-releasing hormone antagonist in the ovariectomized rhesus monkey. *Neuroendocrinology* 64 225–232. 10.1159/000127121 8875440

[B171] Yamamura T. Wakabayashi Y. Ohkura S. Navarro V. M. Okamura H. (2015). Effects of intravenous administration of neurokinin receptor subtype-selective agonists on gonadotropin-releasing hormone pulse generator activity and luteinizing hormone secretion in goats. *J. Reprod. Dev.* 61 20–29. 10.1262/jrd.2014-109 25345909 PMC4354227

[B172] Yang K. Haynes N. B. Lamming G. E. Brooks A. N. (1988). Ovarian steroid hormone involvement in endogenous opioid modulation of LH secretion in mature ewes during the breeding and non-breeding seasons. *J. Reprod. Fertil.* 83 129–139. 10.1530/jrf.0.0830129 3397932

[B173] Yasuda K. Raynor K. Kong H. Breder C. D. Takeda J. Reisine T. (1993). Cloning and functional comparison of κ and δ opioid receptors from mouse brain. *Proc. Natl. Acad. Sci. U. S. A.* 90 6736–6740. 10.1073/pnas.90.14.6736 8393575 PMC47007

[B174] Zheng S. X. Bosch M. A. Ronnekleiv O. K. (2005). μ-opioid receptor mRNA expression in identified hypothalamic neurons. *J. Comp. Neurol.* 487 332–344. 10.1002/cne.20557 15892097

